# Tumor suppressor RARRES1 links tubulin deglutamylation to mitochondrial metabolism and cell survival

**DOI:** 10.18632/oncotarget.26600

**Published:** 2019-02-26

**Authors:** Sara Maimouni, Mi-Hye Lee, You-Me Sung, Michael Hall, Arpita Roy, Chokri Ouaari, Yoo-Seok Hwang, Justin Spivak, Eric Glasgow, Matthew Swift, Jay Patel, Amrita Cheema, Deepak Kumar, Stephen Byers

**Affiliations:** ^1^ Department of Oncology, Georgetown-Lombardi Comprehensive Cancer Center, Georgetown University, Washington, DC, USA; ^2^ Department of Biochemical, Molecular and Cellular Biology, Georgetown University, Washington, DC, USA; ^3^ University of the District of Columbia, Washington, DC, USA; ^4^ Cancer & Developmental Biology Laboratory, National Cancer Institute-Frederick, Frederick, MD, USA

**Keywords:** retinoic acid signaling, RARRES1, metabolic reprogramming, PTM tubulin, drug resistance

## Abstract

RARRES1, a retinoic acid regulated carboxypeptidase inhibitor associated with fatty acid metabolism, stem cell differentiation and tumorigenesis is among the most commonly methylated loci in multiple cancers but has no known mechanism of action. Here we show that RARRES1 interaction with cytoplasmic carboxypeptidase 2 (CCP2) inhibits tubulin deglutamylation, which in turn regulates the mitochondrial voltage dependent anion channel (VDAC1), mitochondrial membrane potential, AMPK activation, energy balance and metabolically reprograms cells and zebrafish to a more energetic and anabolic phenotype. Depletion of *RARRES1* also increases expression of stem cell markers, promotes anoikis, anchorage independent growth and insensitivity to multiple apoptotic stimuli. As depletion of CCP2 or inhibition of VDAC1 reverses the effects of RARRES1 depletion on energy balance and cell survival we conclude that RARRES1 modulation of CCP2-modulated tubulin-mitochondrial VDAC1 interactions is a fundamental regulator of cancer and stem cell metabolism and survival.

## INTRODUCTION

Cancer cells and stem cells share several common features including similar changes in metabolism and resistance to mitochondria mediated cell death [1-3]. Many oncogenic pathways influence these processes, usually indirectly following multistep alterations in metabolic enzymes [4]. There is growing interest in the notion that directly targeting glycolysis hiior mitochondrial metabolic function may influence cancer in much the same way that metformin can be used to treat diabetes [5]. Indeed metformin, a mitochondrial respiration inhibitor, has quite striking anti-cancer activities, although these may involve additional metabolic targets [4]. However, with the exception of LKB1, which regulates the cytoplasmic ATP sensor AMPK, no tumor suppressor genes are known to directly regulate glycolysis or mitochondrial metabolic activity [6, 7].

*RARRES1*, a retinoic acid regulated tumor suppressor gene associated with ageing, metabolism and stem cell differentiation is among the most commonly methylated loci in multiple cancers but has no known molecular function [8-10]. Like LKB1, the *RARRES1* homologue *latexin* is associated with hematopoetic stem cell differentiation and ageing [11, 12]. RARRES1 and latexin are putative carboxypeptidase inhibitors and we showed earlier that RARRES1 interacts with cytoplasmic carboxypeptidase 2 (CCP2/AGBL2 [13]). Both RARRES1 and CCP2 have been associated with metabolic diseases and several studies have identified them as important regulators of autophagy [14-19]. We recently identified RARRES1 as a novel regulator of fatty acid metabolism [20]. CCP2 is a member of the CCP family of deglutamylases important for the removal of glutamic acid residues from the C-terminal tail of several tubulin isoforms [21-24]. Glutamylated and polyglutamylated tubulin is enriched in mitotic spindles and other structures, such as axonemes/cilia that contain arrays of stable microtubules [25, 26]. Although CCPs have not been associated with cancer, the enzymes that modify tubulin (TTL and TTLLs) and detyrosinated tubulin have [24, 27]. Peptide mimics of the acidic C-terminal tail of tubulin can also directly influence the activity of mitochondrial voltage dependent anion channels (VDAC) and mitochondrial membrane potential, raising the possibility that pathways that alter its acidic C-terminal tail could influence mitochondrial activity directly by influencing VDAC function [28-30]. We now show that the metabolic and tumor suppressor effects of RARRES1 are mediated by its inhibition of CCP2 catalyzed tubulin deglutamylation, which in turn regulates mitochondrial bioenergetics and subsequently alters energy homeostasis by modulating the function of the mitochondrial voltage-dependent anion channel 1 (VDAC1).

## RESULTS

### RARRES1, CCP2 and retinoic acid regulate tubulin glutamylation

**Figure 1:**
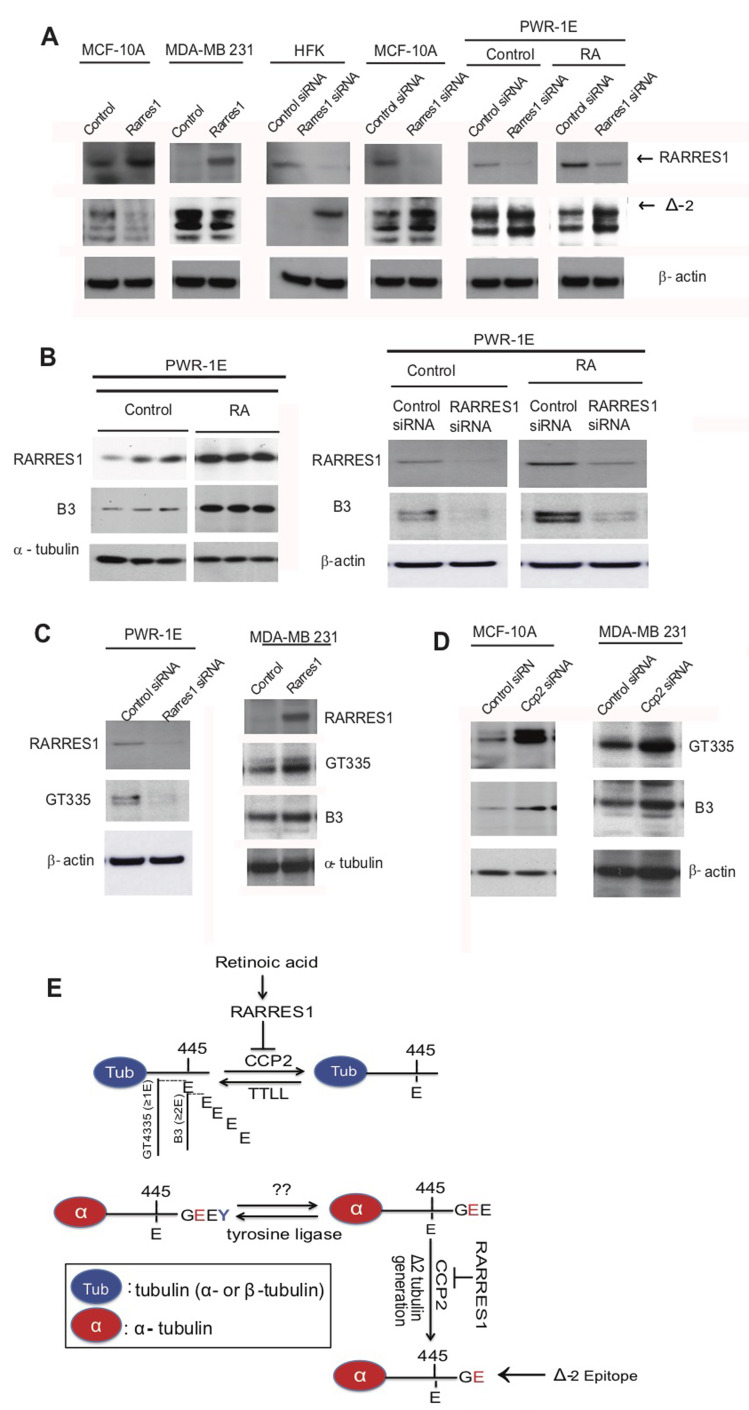
RARRES1, CCP2 and retinoic acid regulate tubulin glutamylation **A.** Δ-2 tubulin levels are regulated by RARRES1 in MDA-10A, MDA-231, HFK and PWR-1E cells. **B.** RARRES1 and polyglutamylated tubulin is increased by retinoic acid (10^-7^M all-trans-RA for 24 hours). Western blots in PWR-1E cells three biological replicates were run for each experimental condition (Vehicle (control) *vs*. RA treatment). RARRES1 siRNA reversed its effect on polyglutamylation. The B3 antibody, which detects tubulin side-chains containing two or more glutamates, was used. **C.** Immunoblot for GT335, which detects one or more glutamates attached to the side chain, was also assessed in PWR-1E cells with RARRES1 knockdown or control knockdown. Immunoblots for GT335 and B3 were done to assess the effects of RARRES1 exogenous expression in MDA-MB-231 cells, in which RARRES1 is silenced. **D.** Immunoblot of CCP2 and polyglutamylated tubulin in CCP2 knockdown MCF10A and MDA-MB-231 cells. **E.** Schematic illustrating the effects of RA and RARRES1 in CCP2 regulation of tubulin deglutamylation. Epitopes of GT335, B3 and ∆-2 antibodies are also shown.

RARRES1 interacts with AGBL2/CCP2 (CCP2), a member of the CCP family of carboxypeptidases responsible for post-translational modifications of the C-terminal region of tubulin [13]. Although CCPs are most commonly associated with ciliated organs, non-ciliated cells exhibit varying glutamylated forms of tubulin and *CCP2* is expressed in many cancer cells [13]. Supplementary Figure 1 shows that several human cancer and normal cells, express significant *CCP2* and demonstrates its successful depletion. However *CCP1*, which is highly expressed in neuronal tissues, was barely detectable in only MDA-MB-231 and BT549 breast cancer cells [31] (Supplementary Figures 1 and 17). *CCP2* has many splice variants, some of which do not contain the catalytic domain (Supplementary Figure 2). The qPCR primers used in this study and our previous work only detect forms of *CCP2* that contain the catalytic domain (Supplementary Figure 2 [13]). CCP2 can remove the penultimate glutamate from tubulin to form Δ2-tubulin, an isoform that can no longer be re-tyrosinated and which accumulates in neurons and in cancer cells [32]. Consequently CCP2 action could indirectly change the relative ratio of tyrosinated and detyrosinated tubulin without actually acting as a detyrosinase [13, 22, 33]. Figure 1 shows for the first time that RARRES1 and its major regulator, retinoic acid (RA), decrease the level of Δ2-tubulin and increase side chain glutamylation of tubulin in primary human keratinocytes and several normal and cancer cell lines by inhibiting CCP2. We selected normal cell lines that endogenously express RARRES1, to perform knockdown experiments. In the case of cancer cell MDA-MB-231, where RARRES1 expression is silenced by methylation, we exogenously express RARRES1 to assess changes in Δ2-tubulin. Importantly the effect of RA on tubulin side chain glutamylation is also dependent upon RARRES1. We used two poly-glutamylated tubulin antibodies, B3, which detects side chains containing two or more glutamic acids and GT335, which recognizes side chains containing one or more glutamic acids [34, 35] (Figure 1B and 1C and Supplementary Figure 3C and 3D). The opposite was seen when RARRES1 was transiently expressed in MDA-MB-231 (Figure 1C). Transient expression of *CCP2* reduced glutamylated tubulin levels and its depletion increased them, consistent with RARRES1 being an inhibitor of CCP2-mediated deglutamylation of tubulin (Figure 1D). Similar results were obtained by immunostaining of cells following RARRES1 or CCP2 depletion (Supplementary Figure 3). These data strongly implicate RARRES1 in the regulation of CCP2-mediated deglutamylation of alpha-tubulin c-termini and of glutamylated side chains (Figure 1E).

### RARRES1 and CCP2 reciprocally regulate sensitivity to a variety of apoptotic stimuli

**Figure 2:**
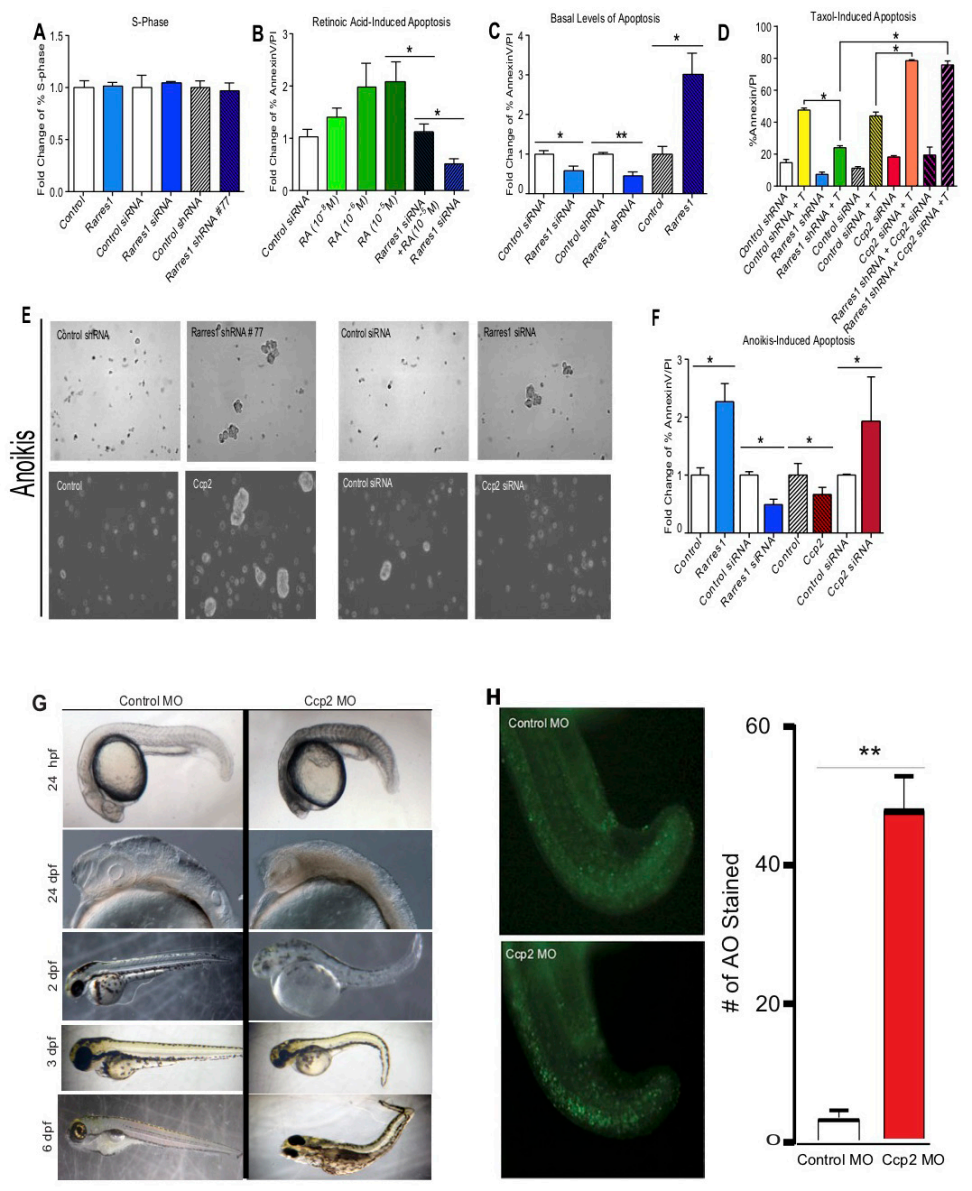
RARRES1 and CCP2 regulate apoptosis but not cell proliferation **A.** Cells were stained with propidium iodide (Sigma) and analyzed by flow cytometry. % S-phase cells are expressed as fold change in MCF10A cells exogenously expressing RARRES1 or following transient or stable knockdown. **B.** RARRES1 depletion inhibits retinoic acid induced apoptosis in MCF10A cells. **C.** RARRES1 exogenous expression, transient and stable depletion, regulate basal levels of apoptosis. **D.** Taxol-induced cell apoptosis. % cell death after RARRES1 or CCP2 manipulation, with, or without taxol (C-control, T-taxol treatment (10ug/ml for 48h). Samples were stained with fluorescein-labeled Annexin V and propidium iodide and analyzed by flow cytometry. **E.** and **F.** RARRES1 and CCP2 have reciprocal effect on anoikis-induced apoptosis. Phase-contrast photographs of cell aggregates/spheroids **E.** and fold change of RARRES1 or CCP2 depleted cells following suspension-induced apoptosis (anoikis) in MCF10A cells **F.**. **G.** CCP2 knockdown causes transient cell death in zebrafish embryos. (1-10] Phenotype following injection of 8 ng control MO [1, 3, 5, 7, 9], or 8 ng agbl2 MO [2, 4, 6, 8, 10]. **H.** Quantification of CCP2 knockdown induced cell death measured by acridine orange staining.

RA is a pleiotropic factor with growth inhibitory, stem cell differentiating and apoptotic properties in normal and cancer cells [36-38]. We next asked if RARRES1 influenced the proliferative and apoptotic effects of RA. MCF10A cells, normal mammary epithelial cells that endogenously express RARRES1 and CCP2, were used as our model. *RARRES1* manipulation did not affect MCF10A cell proliferation or cell cycle but cell death induced by high doses of RA was reversed by depletion of *RARRES1* (Figure 2A, 2B, Supplementary Figure 4). Depletion of *RARRES1* reduced apoptosis and exogenous expression of *RARRES1* enhanced apoptosis even in the absence of RA (Figure 2C). Cell death induced by 48 hour treatment with 10 μg/mL microtubule stabilizing drug taxol was also inhibited by *RARRES1* depletion, while CCP2 knockdown had the opposite effect (Figure 2D). MCF10A cells are exquisitely sensitive to detachment-induced cell death (anoikis) and express both *RARRES1* and *CCP2* (Figure 1 and Supplementary Figure 1, [39]. Anoikis analyses confirmed that most control MCF10A cells died rapidly if they were not attached to a substrate (Figure 2E and 2F). In contrast survival of detached MCF10A cells in which *RARRES1* was stably or transiently depleted was markedly enhanced (Figure 2F). Similar results were observed in MCF10A cells exogenously expressing *CCP2* pointing to a role for RARRES1 inhibition of CCP2-mediated tubulin glutamylation in the regulation of cell death. Detached *RARRES1* depleted cells also formed spheroids while in suspension (Figure 2E). Importantly, in the anoikis and taxol experiments the effects of *RARRES1* depletion were abolished when *CCP2* was also depleted, strongly suggesting that inhibiting CCP2 function mediated the effects of RARRES1 on cell survival in these conditions.

*CCP2* knockdown in zebrafish embryos resulted in transient cell death, slightly stunted embryos but no overt embryological abnormalities (Figure 2G). *CCP2* MO1 was designed to block translation and *CCP2* MO2 to block splicing. Injection of either MO resulted in identical dose dependent phenotypes. *CCP2* knockdown resulted in a subtle but distinct cell-death phenotype. By 24 hpf, tissue became opaque and granular, particularly in the ventral midbrain and hindbrain regions suggesting significant cell-death confirmed by staining apoptotic cells with acridine orange (Figure 2G). Embryos were co-injected with the *CCP2* MO1 and a *tp53* MO since p53 knockdown is known to suppress potential MO induced off-target effects on apoptosis [40]. Counts of acridine orange stained cells at 24 hpf indicated a 10 fold increase in apoptosis in *CCP2* knockdown embryos compared to controls (Figure 2H). This phenotype was transient however, as by 48 hpf, tissues were no longer opaque and granular. Differentiation of the remaining cells appeared to be fairly normal as all tissue types were present, although the embryos were small and somewhat dysmorphic overall (Figure 2G). This stunted phenotype is similar to that previously reported in a smaller number of CCP2 morphants [41]. In our hands this was the dominant phenotype.

### RARRES1 and CCP2 reciprocally regulate anchorage independent growth and are associated with stem cell differentiation

**Figure 3:**
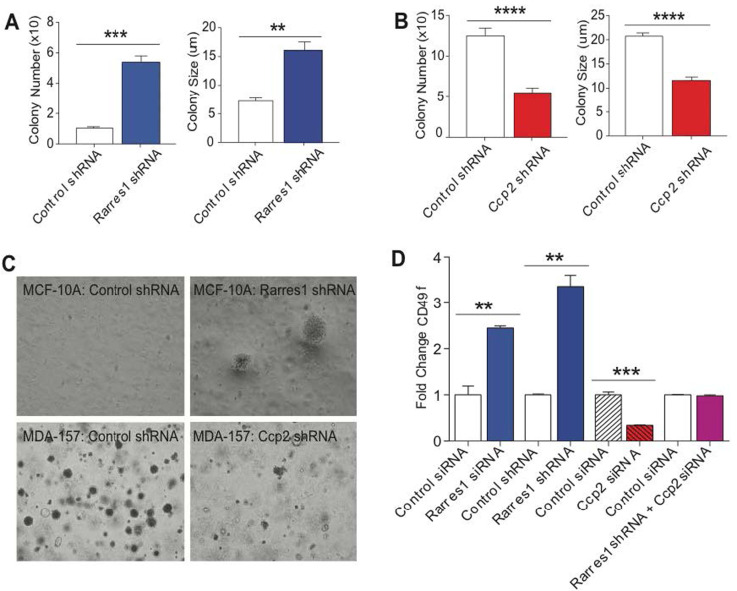
RARRES1 and CCP2 regulate anchorage independent cell growth and CD49f **A.** RARRES1 depletion increases MCF10A colony number and size. **B.** CCP2 depletion decreases MDA-157 colony number and size. **C.** Phase-contrast images of colonies formed following RARRES1 or CCP2 manipulation. **D.** RARRES1 and CCP2 reciprocally regulate stem cell marker, CD49f. Flow cytometry of CD49f labeled cells.

Resistance to anoikis is a requirement for anchorage-independent colony formation, a cardinal indication of transformation and is also a characteristic of many stem cells [42-44]. To test if RARRES1 could regulate colony formation in soft agar we first created stable *RARRES1* knockdown cells (see Materials and Methods). Parental and PLKO control vector MCF10A cells formed very few colonies after 2 weeks in soft agar. Loss of RARRES1 was associated with enhanced colony growth within soft agar (Figure 3A, & 3C). As control MCF10A cells do not form colonies in soft agar we cannot use them to measure inhibition of colony formation. Instead we chose a breast cancer cell MDA157, which forms many colonies in soft agar. These cells express CCP2 but not RARRES1 and stable expression of *RARRES1* in MDA157 and other cells without endogenous RARRES1 causes cell death prior to establishment of stable transfectants. However stable *CCP2* knockdown reduced both the size and number of colonies in MDA157 cells (Figure 3A, 3B & 3C).

RARRES1 and its homologue latexin have been implicated in the regulation of stem cell differentiation and ageing [8, 45, 46]. Breast epithelial stem cells are characterized by, high CD49f (α6-integrin) expression. Consistent with a role in the regulation of stem cell differentiation depletion of RARRES1 from MCF10A cells resulted in a 3-fold increase in cell surface CD49f (Figure 3D, Supplementary Figure 5). Conversely, depletion of *CCP2* reduced CD49f cell surface expression and completely prevented the stimulatory effects of *RARRES1* depletion. Taken together these data demonstrate that RARRES1 and CCP2 reciprocally regulate, expression of CD49f, anoikis, anchorage independent growth and sensitivity to a variety of apoptotic stimuli in cells and zebrafish. Moreover depletion of *CCP2* reversed the effects of *RARRES1* depletion consistent with a role for RARRES1 as an inhibitor of CCP2 function. As CCP2 is a tubulin deglutamylase and RARRES1 both interacts with CCP2 and regulates tubulin glutamylation these data are consistent with a novel role for tubulin glutamylation in stem cell differentiation and the regulation of cell survival. Several studies have pointed to an association of glutamylated tubulin, tubulin glutamylases (TTLLs) and the CCP2 product ∆2-tubulin with cancer and resistance to treatment, but the molecular mechanism(s) or the result of these associations is completely unknown [23, 24]. In other studies we showed that RARRES1 controls fatty acid and glucose metabolism by regulating the switch from aerobic glycolysis to glucose dependent de novo lipogenesis [20]. Taken together with its effects on apoptosis shown here and elsewhere, these data point to a role for RARRES1-mediated changes in tubulin glutamylation (both C-terminal and side chain) in the general regulation of metabolism and mitochondrial function. Consistent with this possibility are studies that show that RARRES1 and post-translational modification of tubulin can regulate lipid metabolism and the transport of lipid droplets to the mitochondria respectively [20, 47, 48]. Importantly, biophysical studies show a marked effect of BSA conjugated to tubulin-like C-terminal peptides on the activity of the mitochondrial voltage dependent anion channel (VDAC), an important regulator of mitochondrial-dependent cell death, and mitochondrial membrane potential. This activity is reversed when both the C-terminal tyrosine and the penultimate glutamic acid are removed to form a ∆2-tubulin-like C-terminal or if cells are treated with erastin, which displaces tubulin from VDAC [51]. These studies complement other studies that show certain isoforms of tubulin are often found associated with mitochondria [49]. The regulation of the VDAC is likely to influence oxidative phosphorylation and energy balance, functions that are important in stem and cancer cell function as well as resistance to apoptosis. As the pleiotropic effects of RARRES1 involve the regulation of tubulin C-terminal modifications by CCP2 (including the formation of ∆2-tubulin) we next asked if RARRES1 could alter mitochondrial membrane potential.

### RARRES1 regulates mitochondrial membrane potential (MMP)

**Figure 4:**
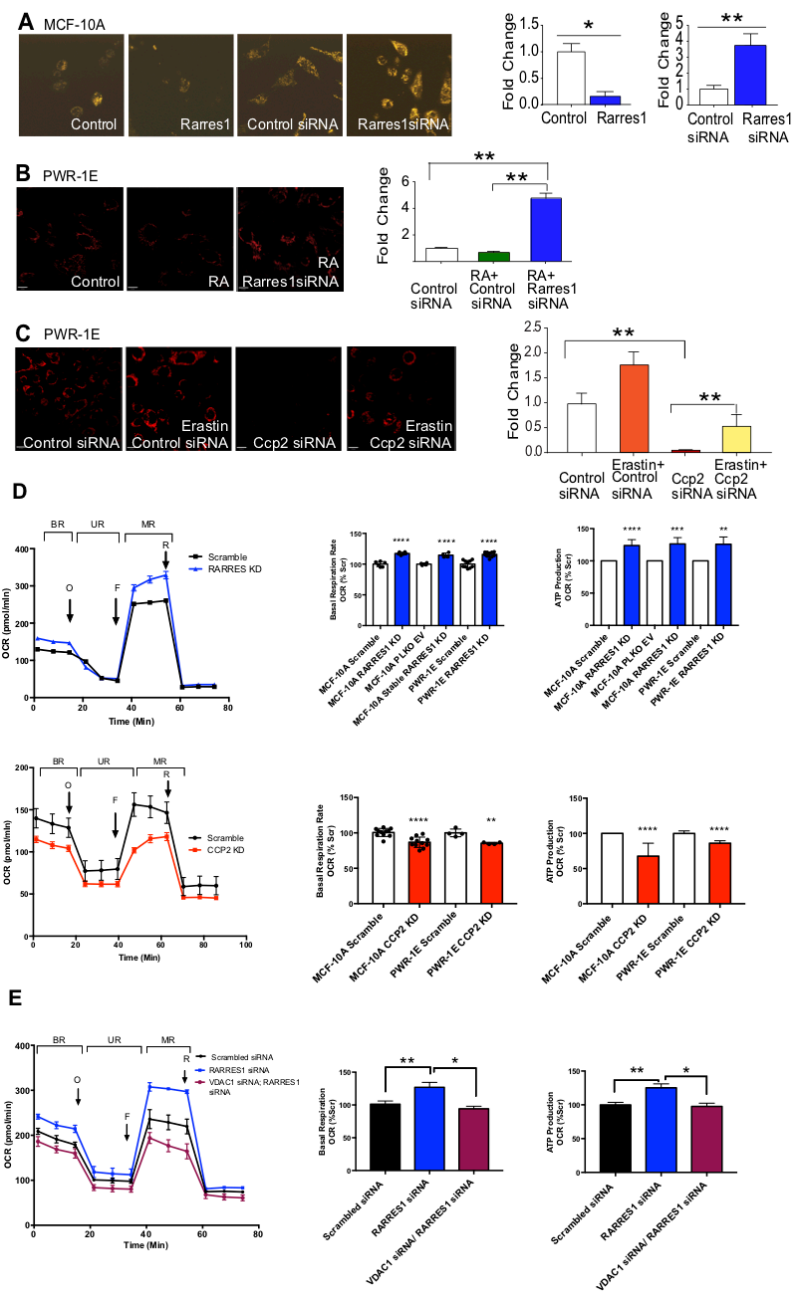
RARRES1 and CCP2 regulate Mitochondrial Membrane Potential (MMP) and mitochondrial respiration **A.-C.** MMP measurement by TMRM assay after RARRES1/CCP2 manipulation, retinoic acid or erastin treatment, in MCF10A and PWR-1E cells. Fluorescent images of cells treated with the indicated reagents. Integrated brightness of TMRM assay measured by Keyence analysis software and converted to fold change. **D.** Oxygen consumption rate (OCR) of RARRES1 depleted MCF10A and PWR-E cells profiled in the extracellular flux assay. The relative quantification of the area below the curves corresponding to the stages labeled as BR (Basal Rate), UR (Uncoupling Respiration Rate), and MR (Spare Respiratory Capacity) are shown as histograms. OCR in PWR-1E and MCF10A cells following transient CCP2 depletion. **E.** RARRES1 depleted MCF 10A cells were also simultaneously transfected with VDAC1 siRNA. OCR was measured in all experimental groups.

MMP like other aspects of mitochondrial function is finely tuned to the proliferative and energetic status of the cell; VDACs that are too active might lead to dangerous levels of reactive oxygen species, VDACs that are not active enough could lead to a change in ATP/ADP ratio and a switch to aerobic glycolysis (the Warburg effect). We wondered if the effects of RARRES1 on tubulin glutamylation might contribute to the regulation of the VDAC and influence MMP. To test this we first imaged *RARRES1* and *CCP2* manipulated cells with mitotracker. This dye changes color depending on the pH of the mitochondrial lumen and provides an indirect (and approximate) assessment of MMP. Supplementary Figure 6A-6D shows that alterations in mitochondrial pH (and morphology) take place in *RARRES1* depleted cells. To further investigate this we labeled cells with the MMP-sensitive dye TMRM and quantified the effects of *RARRES1* depletion on MMP (Figure 4A-4D and Supplementary Figure 6E). These data confirm that depletion of *RARRES1* significantly increased MMP likely indicative of an increase in oxidative phosphorylation. RA treatment also decreased MMP in a RARRES1 dependent manner. In contrast *CCP2* depletion reduced MMP and its exogenous expression increased it. To confirm that RARRES1/CCP2 regulation of MMP involved VDAC we used erastin. Although originally discovered in a screen for inhibitors of ras signaling, erastin is known to be a VDAC interactor that displaces acidic tubulin-like peptides from the VDAC [50-52]. Co-treatment with erastin significantly inhibited the decrease in MMP following *CCP2* depletion providing evidence that the effects of CCP2/RARRES1 on MMP are mediated by regulation of VDAC activity by tubulin glutamylation (Figure 4D).

### RARRES1 and CCP2 control mitochondrial respiration by regulation of VDAC1-mediated intracellular substrate availability

To determine whether RARRES1 and CCP2 regulate mitochondrial activity and glycolytic potential, they were silenced and oxygen consumption rates (OCR) and extracellular acidification rates (ECAR) analyzed. We were initially concerned that any data we generated from *RARRES* depleted stable cell lines may reflect an indirect, adaptation response to long term *RARRES1* knockdown. This was not the case however, as in all experiments the results from either stable or transient knockdowns were similar. Silencing *RARRES1* in both MCF-10A and PWR-1E cells improved their energetic status (Figure 4E). Basal OCR increased suggesting that *RARRES1* depleted cells rely more on mitochondrial respiration and increased ATP-turnover (Figure 4E). The spare respiratory capacity increased, which indicates higher substrate availability with which to conduct mitochondrial respiration and electron transport chain activity. The uncoupling response (UR) OCR, measured after oligomycin injection, was significantly increased compared to controls and indicates an increase in ATP production. To confirm that the interaction of RARRES1 with CCP2 contributes to its effects on mitochondrial respiration, we depleted MCF10A and PWR-1E cells of *CCP2* (Figure 4E). Consistent with a role for RARRES1 as an inhibitor of CCP2, *CCP2* knockdown decreased ATP production and basal respiration rate and resulted in an overall decrease in the energetic status of cells (Figure 4E). RARRES1 depletion could not rescue the effects of CCP2 knockdown in cells (Supplementary Figure 7A) indicating that the effect of RARRES1 on mitochondrial respiration is a consequence of its regulation of CCP2, as it was in cell survival (Figure 2F).

We next confirmed that the effects of CCP2/RARRES1 on mitochondrial metabolism required VDAC. We focused on VDAC1 since it is the dominant isoform in our cell models. The increase in OCR that occurs when RARRES1 is depleted was reversed when both RARRES1 and VDAC1 were simultaneously depleted in either MCF 10A cells or PWR-1E cells (Figure 4F and Supplementary Figure 7B). Thus, the effects of RARRES1 and CCP2 on mitochondrial respiration are due to regulation of VDAC1 activity.

### RARRES1 and CCP2 reciprocally regulate glycolytic demand

**Figure 5:**
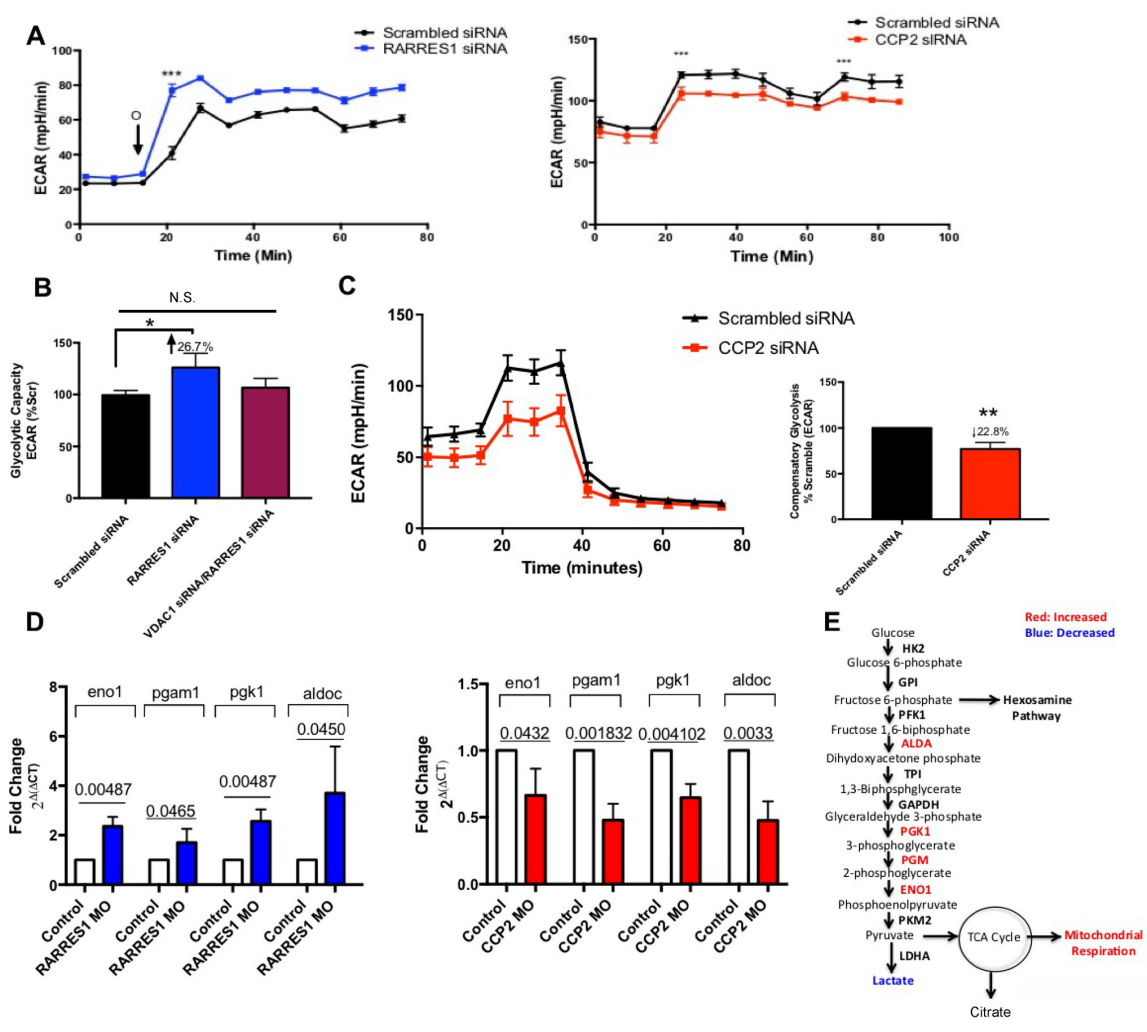
RARRES1 depleted cells are less dependent on glycolysis **A.** RARRES1 and CCP2 depletion alters ECAR response to inhibition of mitochondrial respiration by oligomycin. **B.** ECAR response to inhibition of mitochondrial respiration in VDAC1 and RARRES1 double knockdown MCF 10A cells was assessed. **C.** PWR-1E cells transfected with scrambled siRNA or CCP2 siRNA were subjected to glucose prior to the assay and a combination of antimycin A and rotenone and 2-deoxy-d-glucose were injected. The compensatory glycolysis rate was assessed. **D.** Enzymes related to glycolysis were assessed in RARRES1 MO and CCP2 MO zebrafish. The transcript levels of these genes were quantified using qPCR. **E.** Enzymes, byproducts and pathways related to glycolysis that increased (highlighted in red) or decreased (highlighted in blue) following RARRES1 depletion.

Hexokinase II (HKII) can bind to VDAC1 and alter coupling between mitochondrial respiration and glycolysis [53, 54]. The binding of HKII to VDAC1 facilitates ATP accessibility to HKII and helps it catalyze the first reaction in glycolysis [55, 56]. We showed previously that RARRES1 depleted cells redirect glucose for fatty acid synthesis [20]. In RARRES1 depleted PWR-1E and MCF 10A cells a significant increase in glycolytic capacity or compensatory glycolysis accompanied the ECAR shift that occurred after inhibition of mitochondrial respiration. Here we wanted to test if the effects of RARRES1 on glycolytic capacity are a result of its modulation of tubulin blockade of VDAC1. We thus assessed whether the target of RARRES1, CCP2, can regulate coupling between glycolysis and oxidative phosphorylation. We monitored the glycolytic shift that occurs following inhibition of mitochondrial respiration, a phenomenon called glycolytic capacity or compensatory glycolysis. Uncoupling respiration with oligomycin increased the ECAR indicating an improved glycolytic capacity in *RARRES1* knockdown cells, as observed in our previous study [20]. The opposite is seen in CCP2 depleted cells (Figure 5A). To confirm that the ECAR shift seen in RARRES1 depleted cells is mediated through VDAC1 we transiently depleted VDAC1 in RARRES1 depleted cells and measured the ECAR shift after oligomycin treatment. The double knockdown reversed the effects of RARRES1 depletion, and the significant ECAR increase seen in RARRES1-depleted cells was no longer observed. Thus the glycolytic coupling seen in RARRES1 depleted cells is mediated through modulation of VDAC1 activity (Figure 5B). To ensure that the shift in ECAR is due to glycolysis, we examined glycolytic activity of CCP2 depleted cells using the glycolytic rate assay as well as the glycolysis stress test. The glycolysis stress test starves cells of glucose prior to the assay; glucose is subsequently injected during the run, which enables the measurement of the real-time conversion of glucose into lactate (Supplementary Figure 8). The glycolytic rate assay measures glycolysis-dependent ECAR without glucose starvation prior to the assay. Therefore, this assay measures basal levels of glycolysis without perturbing the metabolic needs of the cells. We used both assays to ensure CCP2 affects glycolysis during glucose starvation and in normal conditions. We found that CCP2 depletion caused a significant decrease in glycolytic capacity or compensatory glycolysis in the two different conditions (Figure 5C and Supplementary Figure 8).

To further examine a role for RARRES1 and CCP2 in glycolysis, we performed knockdown studies in zebrafish embryos and monitored levels of enzymes important in glycolysis. Knockdown of *RARRES1* increased levels of enolase-1 (eno1), phosphoglycerate mutase 1A (pgam1), phosphoglycerate kinase 1 (pgk1), and aldolase c (aldoc) (Figure 5D & 5E). In contrast, knockdown of *CCP2* decreased levels of these enzymes. These reciprocal changes in the levels of enzymes important in metabolism indicate a function for RARRES1 in central carbon metabolism and points to a role for the RARRES1/CCP2 axis in the regulation of coupling between oxidative phosphorylation and glycolysis.

### RARRES1 and CCP2 regulate energy homeostasis

**Figure 6:**
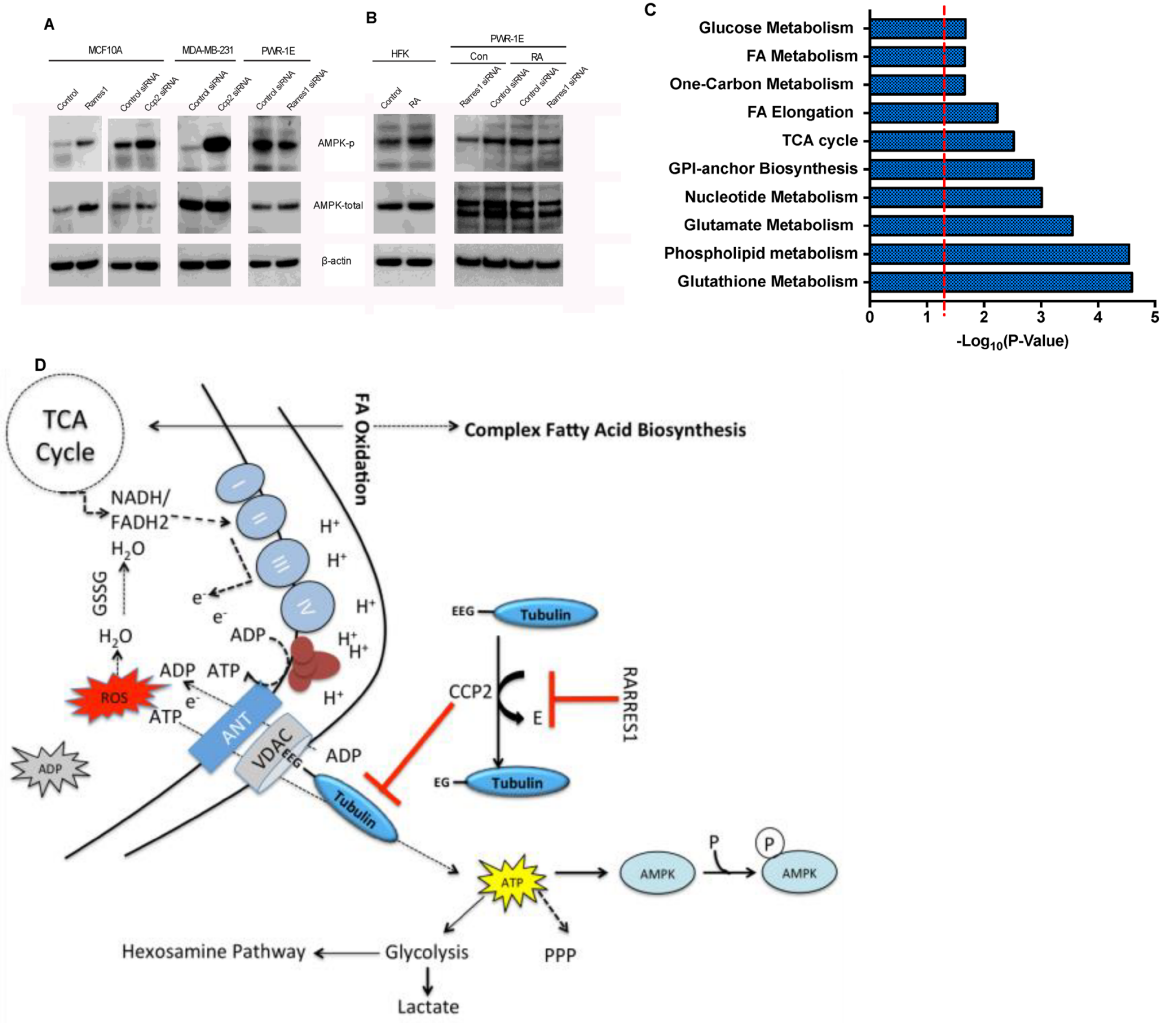
RARRES1 regulates energy metabolism **A.** Immunoblot for total and phosphorylated AMPK in MCF10A, MDA-MB-231 or in which RARRES1 or CCP2 was exogenously expressed or depleted. Refer to Supplementary Figure 16 for full-length blots. **B.** Immunoblot for total and phosphorylated AMPK HFK and PWR-1E cells with or without RA after RARRES1 depletion. **C.** Enriched pathways in RARRES1 stable knockdown MCF10A cells. The dashed line represents *p* = 0.05. **D.** Schematic of the biological effects of RARRES1 and CCP2.

VDAC1 regulates ATP/ADP release from the mitochondria. VDAC inhibition by itraconazole and knockout studies have shown that VDAC1 regulation of ATP/ADP transport regulates ATP availability in the cytoplasm and subsequently dictates AMP kinase activity [50]. AMP kinase is a central regulator of metabolic reprogramming and is sensitive to nutrient availability and the AMP/ATP ratio [57]. Starvation or increased AMP results in phosphorylation and activation of AMP kinase by liver kinase B1 (LKB1) [58]. Since RARRES1 and CCP2 govern mitochondrial energetics through VDAC1, we examined whether they can affect AMP kinase activity. Levels of activated AMPK increase significantly upon exogenous expression of *RARRES1* or depletion of *CCP2* and decreased upon *RARRES1* depletion (Figure 6A). The RARRES1 inducer retinoic acid also activated AMP kinase in a RARRES1-dependent manner in both HFK and PWR-1E cells (Figure 6B). Interestingly, exogenous expression of RARRES1 in MCF10A cells increased both protein and phosphorylation levels of AMP kinase. Since this was only seen in one of our cell lines, we believe this could be a cell specific response rather than a biologically relevant phenomenon.

AMPK directly phosphorylates a number of enzymes that mediate metabolism and growth, such as acetyl-coA carboxylase 1 and 2 and 6-phosphofructo-2-kinase/fructose-2,6-bisphosphatase 2/3. Generally, AMPK activation rewires metabolic pathways to promote energy production by inducing catabolic pathways, such as glycolysis and fatty acid oxidation, while inhibiting anabolic pathways such as fatty acid and protein synthesis [58]. We showed previously that RARRES1 depletion increases de novo lipogenesis, one of the pathways inhibited by AMP kinase. In this study we examined whether other pathways regulated by AMP kinase are also altered in RARRES1 depleted cells. Since RARRES1 depletion decreases AMP kinase activity, we hypothesized that RARRES1 depletion would reprogram cells to a more anabolic state. We validated and measured significantly altered metabolites in the RARRES1 depleted MCF 10A cells using primary data from our previous work [20] (Supplementary Table 1). Many metabolites participating in central carbon metabolism were altered in the *RARRES1* knockdown. Pathway enrichment analysis identified a number of metabolic pathways that were significantly changed including; the citric acid cycle, glycolysis, fatty acid, glutamate and glutathione metabolism (Figure 6C & Supplementary Table 1). *RARRES1* knockdown increased oxidized glutathione suggesting a cellular response to high oxidative stress induced by *RARRES1* knockdown. We showed previously that basal glycolysis is decreased in *RARRES1* depleted cells although their glycolytic reserve increased and that RARRES1 depletion redirected glucose metabolism for lipid synthesis. In our non-targeted LC-MS analysis of stable RARRES1 knockdown, we also observed a 7-fold increase in UDP-N-acetylglucosamine. This suggests that part of the glycolytic reserve is being used for the production of UDP-N-acetylglucosamine, the end product of the hexosamine pathway, a minor branch of glycolysis where fructose-6-phosphate is converted to glucosamine-6-phosphate, catalyzed by fructose-6-phosphate amidotransferase (Supplementary Table 1 & Figure 6C). We also observed a 16 fold-change in UDP-glucose, a precursor of glycogen (Supplementary Table 1). Glycogenesis is another branch of glycolysis in which glucose is converted to glucose-6-phosphate and subsequently catabolized to glycogen. The large (> 50 fold) increase of glutathione along with UDP-glucose and UDP-galactose also indicate an increase in anabolic pathways such as glycogenesis and the pentose phosphate pathway (Supplementary Table 1). Taken together these data support the MMP, energy flux and AMP kinase activity assays, and indicate that *RARRES1* knockdown increased mitochondrial respiration and ATP production to drive essential anabolic reactions by attenuating AMP kinase activity (Figure 6D).

### Inhibition of VDAC1 activity re-sensitizes RARRES1-depleted cells to apoptosis

**Figure 7:**
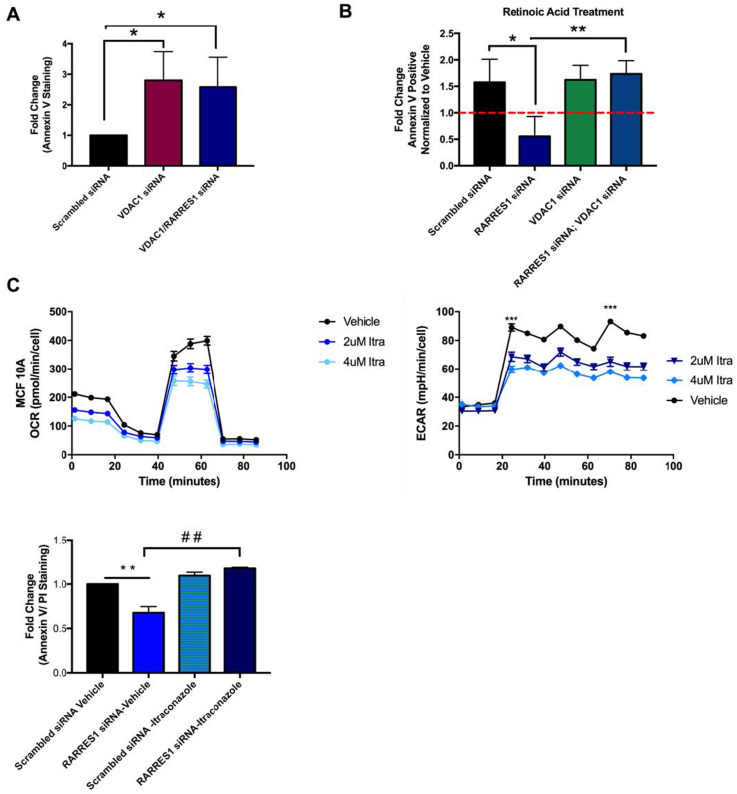
VDAC1 depletion or inhibition reverses the apoptotic phenotype seen in RARRES1-depleted cells **A.** Flow cytometry was conducted. Cells were stained with Annexin V and propidium iodine (PI). VDAC1 depletion induced apoptosis and RARRES1 depleted cells are sensitive to VDAC1 depletion. **B.** MCF 10A cells were treated with retinoic acid or vehicle and subjected to annexin V and PI staining and subsequent flow cytometry analysis. The retinoic acid treated cells were normalized to vehicle. RARRES1-depleted cells were resistant to apoptosis whereas RARRES1-depleted cells with VDAC1 depletion, were sensitive to retinoic acid treatment (Supplementary Figure 8). **C.** MCF 10A cells were treated with 2 μM and 4 μM for 24 hours and OCR and ECAR were profiled in the extracellular flux assay using the Cell Mito Stress Test. Lower panel- 4 μM of itraconazole or vehicle treated scrambled siRNA or RARRES1 siRNA transfected MCF 10A cells. Cells were stained with Annexin V and PI and analyzed by flow cytometry. Itraconazole induced apoptosis and reversed the pro-survival effects of RARRES1 depletion.

Our data shows that RARRES1 and CCP2 reciprocally regulate metabolism through their modulation of VDAC1 activity. However these data do not address whether their effects on VDAC1 activity also mediate their effects on cell survival. Therefore to test if the effect of RARRES1 on cell survival involves VDAC1, we assessed the “resistant” phenotype of RARRES1-depleted cells when VDAC1 is also depleted or inhibited. Apoptosis resistant RARRES1-depleted cells were re-sensitized to apoptosis when VDAC1 was also depleted (Figure 7A, Supplementary Figure 9A). RARRES1 depletion decreased the rate of RA-induced apoptosis but when VDAC1 was depleted in these cells, the cells were resensitized to apoptosis (Figure 7B, Supplementary Figure 9A). This clearly indicates that the RARRES1 effect on cell survival is mediated through VDAC1. We next used itraconazole, an anti-fungal drug that binds to and inhibits VDAC1 in mammalian cells [50]. We first assessed the effects of itraconazole on mitochondrial respiration and glycolytic coupling (Figure 7C). Itraconazole had the same effects on mitochondrial respiration as CCP2 siRNA (see Figures 4E, 5A, and 7B) and reversed the effects of RARRES1 depletion on apoptosis (Figure 7C, Supplementary Figure 9B).

## DISCUSSION

Although no molecular mechanism has been characterized for the pleiotropic effects of RARRES1 on cells and cancers of multiple origins, we recently demonstrated that RARRES1 is a novel modulator of glucose dependent *de novo* lipogenesis and modulates expression of major metabolic regulators, mTOR and SIRT1 [17, 20]. We also showed previously that RARRES1 interacts with CCP2, a tubulin C-terminal deglutamylase responsible for the production of ∆2-tubulin [13, 22]. However, it was not clear how this activity was linked to the anti-cancer or metabolic effects of RARRES1. Post-translational modifications of the acidic C-terminal tail of tubulin have long been linked with modulation of microtubule function [59]. These modifications included glutamylation, tyrosination and glycylation and are often associated with microtubule activities in cilia, spindles and other stable microtubule arrays associated with mitosis. Although anti-microtubule drugs are effective anti-cancer agents there is growing evidence that their primary therapeutic action does not involve direct effects on mitosis (reviewed in [60]). Consistent with this view RARRES1 and CCP2 manipulation does not change cell division (Figure 2A). Tubulin-like C-terminal peptides can interact with the mitochondrial voltage-dependent anion channel (VDAC) to influence mitochondrial membrane potential [30]. Here we propose a novel model for the action of a tumor suppressor and metabolic regulator gene. Our results show that tumor suppressive and metabolic effects of a type II tumor suppressor (the carboxypeptidase inhibitor RARRES1 in this case) can be mediated by inhibition of CCP-catalyzed tubulin C-terminal deglutamylation, which in turn directly regulates VDAC1 activity, mitochondrial membrane potential (MMP), reprograms intermediary metabolism and influences cell survival (Figure 6D). RARRES1 can also affect tubulin side chain glutamylation and may influence lipid droplet transport and metabolism [20, 47, 48]. Taken together these data indicate that this RARRES1 regulation of mitochondrial function may be particularly important in the action of RA as well as the survival and drug resistance of cancer stem-like populations [3, 61].

The simplest molecular explanation consistent with our results is that high CCP2 activity removes glutamic acid residues from the tubulin C-terminal prevent it from attenuating VDAC activity. RA through RARRES1 blocks CCP2 to inhibit deglutamylation, inhibit VDAC, reduce MMP and activate AMPK (Figure 6D). Reduction in RARRES1 increases MMP and oxidative phosphorylation, which in turn alters ADP/ATP flux, and inactivates AMPK with concomitant changes in multiple metabolic and transcriptional pathways to result in a more energetic and anabolic phenotype. VDAC1 depletion reversed the apoptotic and metabolic phenotype of RARRES1 depleted cells. Consistent with this, the *RARRES1* gene is heavily methylated in multiple cancers. Both tubulin and VDAC1 are highly abundant proteins although their exact stoichiometry is not known. However it is clear that most mitochondrial VDACs normally exist in the closed state, consistent with the high concentrations of tubulin available to interact with them [62]. Consequently, small, localized changes in VDAC permeability following CCP2-mediated deglutamylation may have significant effects on anion transport and MMP. Future studies will be directed to distinguishing the relative contribution of RARRES1 regulated tubulin side chain and C-terminal glutamylation on cell survival and metabolism.

We recognize that our data is not consistent with the Warburg model in which cancer cells are hypothesized to reduce their dependence on oxidative phosphorylation and increase aerobic glycolysis [63]. However the metabolic and phenotypic changes induced by *RARRES1* depletion are characteristic of proliferative stem cells and likely activated cancer initiating cells [64]. The changes we observed are also consistent with the metabolic reprogramming that occurs following anchorage independent growth and the switch from quiescent to proliferating mammary cells [65, 66]. The elevated OCR we observed following *RARRES1* depletion might be expected to increase the production of ROS and our LC-MS studies demonstrate an increase in protective ROS scavengers in *RARRES1* depleted cells. Increased ROS following increased mitochondrial respiration also inhibits glyceraldehyde-3-phosphate dehydrogenase (GAPDH) to activate glycogenesis and hexosamine pathways [67]. Both pathways are increased by *RARRES1* depletion, suggesting that increased oxidative phosphorylation decreased AMP kinase activity, which in turn decreased anaerobic glycolysis and shifted glucose metabolism toward biosynthetic pathways.

*RARRES1*/CCP2 also regulates coupling between glucose and mitochondrial metabolism. Consistent with a role for RARRES1/CCP2 in regulating VDAC function, VDAC1 is also an important regulator of the coupling between glycolytic and mitochondrial metabolism. Hexokinase II (HKII) localizes to the VDAC/adenine nucleotide translocase channel where it stimulates the rapid phosphorylation of glucose using ATP that is translocated directly from the mitochondria [56]. HKII also enhances ADP recycling between the cytoplasm and the mitochondria potentially explaining how RARRES1 loss increased mitochondrial respiration as well as glycolytic capacity while CCP2 had the opposite effect [55]. VDAC1 depletion in RARRES1 depleted cells was able to reverse the increase in glycolytic capacity. Taken together, these data support the concept that increased VDAC activity as a result of *RARRES1* depletion enhances the coupling of HKII and ATP synthase, while CCP2 depletion displaces HKII from VDAC1 by mediating tubulin dependent blockage of the channel.

**Figure 8:**
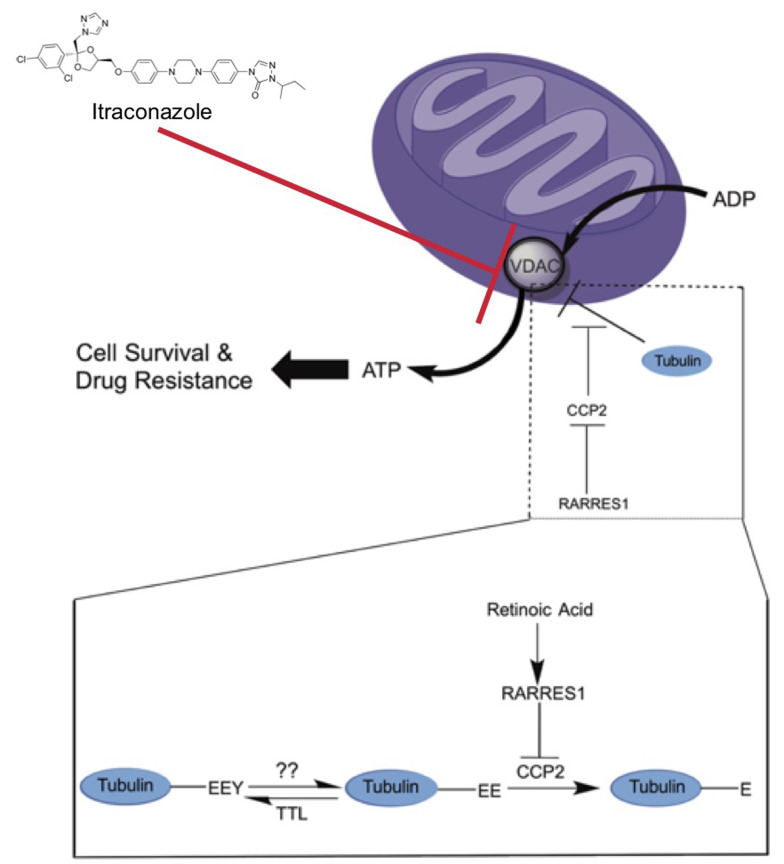
Schematic diagram of the effects of RARRES1 and CCP2 on post translational modification of tubulin and the mitochondrial voltage dependent anion channel

Our data is consistent with growing evidence that stem cells and cancer initiating cells exhibit quite different metabolic characteristics than their differentiated/tumor counterparts [61]. Loss of *RARRES1* in multiple tumor types, its role in stem cell differentiation and in cell survival indicates that the RARRES1/CCP2 axis could be a candidate for therapeutic intervention (Figure 8). In the many cancers in which the *RARRES1* gene is methylated, the unencumbered enzymatic activity of CCP2 could be inhibited by, new or repurposed drugs. Understanding how *RARRES1* expression is regulated could lead to interventions that increase its levels. Alternatively, one could imagine that agents that mimic the effects of RARRES1 (i.e. inhibit mitochondrial respiration) would reverse the effects of RARRES1 loss. In this study, we were able to successfully reverse the resistant phenotype of RARRES1-depleted cells by treating cells with itraconazole, an anti-fungal drug that inhibits the activity of VDAC in mammalian cells. Finally, in an age of deep sequencing and personalized medicine our data support the notion that treatments that target fundamental cell functions such as, metabolism, immune rejection, DNA replication and mitosis, have an important role in the treatment of drug resistant cancers characterized by multiple mutations or translocations and/or a proliferative stem-like phenotype.

## MATERIALS AND METHODS

### Cell culture

PWR-1E, MCF10A, MDA-MB-231, MDA-MB-157, Human neonatal foreskin primary epidermal keratinocyte (HFK) cells were maintained according to the recommendation of American Type Culture Collection. Human pancreatic stellate cells (HPaSteC) were cultured and maintained in Stellate Cell Medium (ScienceCell Research Laboratories) according to supplier’s directions. The passage numbers of all cell lines were below 30 between thawing and use in the experiments. All cell lines were authenticated and tested for mycoplasma by the Lombardi Comprehensive Cancer Center Tissue Culture Shared Resources.

### Antibodies and reagents

All-*trans*-RA and Erastin were obtained from Sigma-Aldrich (St. Louis, MO). Primary antibodies targeting the following antigens were used: rabbit anti-human RARRES1, mouse B3-polyglutamylated tubulin, mouse anti-β actin, and mouse anti α-tubulin were from Sigma-Aldrich and GT335-polyglutamylated tubulin was from Adipogen and D-2 tubulin antibody was from Upstate. Phosphorylated AMPK and total AMPK antibodies were from Cell Signaling. Western blots and immunofluorescent staining were performed as described previously [39]. Full-length western blots are displayed in Supplementary Figures 9–16.

### siRNA, expression constructs, and transfection

Codon-optimized full-length human RARRES1 cloned into *Bgl*II and *Hind*III sites in the pEYFP-N1 vector (Clonetech) and pGlue vector were provided by Genscript. AGBL2 (CCP2; Myc-DDK-tagged) construct was from Origene Technologies, Inc. (Cat #RC206949). ON-TARGETplus SMARTpool Human RARRES1 (Cat #L-009925-00), siGENOME SMARTpool Human AGBL2 (CCP2) (Cat #M-012937-00) and Non-Targeting control siRNA (Cat #D-001210-01-20) were from Thermo Scientific Dharmacon. Cells were seeded at 100,000 cells per well in 6-well plastic tissue culture dishes on day 0. On day 1, for each cell sample, 7.5μl of Lipofectamine 3000 (Invitrogen, Carlsbad, CA) was added to 125μl of OPTI-MEM I (Gibco-Invitrogen, Grand Island, NY) in a sterile eppendorf tube, and mixed. 2.5μg of DNA or 75pmol of siRNA in 125ul of OPTI-MEM were added with or without 5ul of P3000, mixed, and incubated for 5 minutes at room temperature. The mixture was then added dropwise to cells.

### RARRES1/CCP2 shRNA

Constructs for stable depletion of RARRES1 and CCP2 were obtained from the RNAi Consortium (Moffat et al., 2006) *via* SIGMA-Aldrich (NM_002888 for RARRES1 and NM_024783 for CCP2). Five pre-made constructs were obtained for both RARRES1 gene (Clone ID: TRCN0000063373, TRCN0000063374, TRCN0000063375, TRCN0000063376, TRCN0000063377) and CCP2 gene (Clone ID: TRCN0000073908, TRCN0000073909, TRCN0000073910, TRCN0000073911, TRCN0000073912) and individually tested to identify those able to achieve efficient knockdown at the protein level. Negative control constructs in the same vector system (vector alone, pLKO.1 puro) were obtained from SIGMA-Aldrich (NM_003177).

### Cell cycle analysis and flow cytometry

Cells were fixed in 70% ethanol and stained in PBS containing 0.1% Triton X-100, 50 μg/mL RNase, and 50 μg/mL propidium iodide. Samples were analyzed by, flow cytometry and the hypodiploid peak constituted the apoptotic population in this analysis. DNA content was measured on a FACSort flow cytometer (Becton Dickinson, Franklin Lakes, NJ), and data were analyzed using ModFit software (Verity Software House, Topsham, ME). At least 1x10^6^ cells were analyzed per sample. For Annexin V Labeling, samples were stained with fluorescein-labeled Annexin V and propidium iodide (Trevigen) according to the manufacturer’s protocol. The two Annexin V positive quadrants of the analysis were taken as the apoptotic fraction. Anoikis Assays were carried out as described previously [39]. To perform the analysis of cell surface markers (CD44+/CD24-, CD49+/CD24-), MCF10A cells were detached with trypsin, washed in blocking buffer (PBS containing 3% FBS), then stained with anti-CD24-PE (BD Biosciences, San Diego, CA, USA) and anti-CD44-PE-Cy5 (BD Biosciences) or anti-CD49-FITC (BD Biosciences) using 1 ml of antibody per 10^6^ cells, and incubated at room temperature for 1 hr. Following incubation, cells were washed twice with 1 ml PBS. Cells were re-suspended in 1 ml PBS and then analyzed.

### Soft agar growth assay

5,000 cells were plated in 0.3% agar layered on top of 0.6% agar in 6 well plate. After 2 weeks, colonies were stained with 0.005% crystal violet and pictures taken to count and measure colonies size using MetaMorph Image analysis software 7.0 [39].

### Zebrafish animal model

Zebrafish (*Danio rerio*) were raised, maintained and crossed as described previously [68]. Development of embryos was at 28°C and staging was determined by both hpf and morphological characteristics [69]. For morpholino oligonucleotide (MO) injection experiments, two MOs were used to knockdown CCP2 (Agbl2); *abgl2* MO1 was designed to block translation, and, *agbl2* MO2 was designed to block splicing. The following MOs were synthesized by Gene-Tools, LCC: *agbl2* MO1, 5’-AAGGATCGTCTGATTTCTTCTGCAT; *agbl2(CCP2)* MO2, 5’-CCGTGTTGTCATTATAAATGAACGC; and *tp53* MO, 5’-GCGCCATTGCTTTGCAAGAATTG. The Standard Control MO from Gene Tools was used as control. Solutions consisting of 4 ng/nl MO plus 0.5% tetramethyl rhodamine dextran in dH20 were microinjected into one to four cell stage embryos. Acridine orange staining was done as previously described [70].

### Mitotracker and TMRM assay

For Mitotracker staining, cells were seeded at 1X10^5^ and grown on glass coverslips for 24 hours. 24 or 48 hrs post-transfection, cells were stained with Mitotracker Deep Red FM reagent from Life Technologies and more detailed the procedures were followed according to supplier’s directions. 24 or 48 hrs post-transfection, Tetramethylrhodamine methylester (TMRM), a lipophilic cationic fluorescent redistribution dye was applied to detect the mitochondrial membrane potential (D) from MitoPT TMRE & TMRM Assay Kits (ImmunoChemistry Technologies). Cells in complete growth medium were loaded for 20 min at 37°C with 70 nM of TMRM and shaken. More detailed procedures followed the supplier’s directions. TMRM-loaded cells were incubated in 1x Assay Buffer in humidified 5% CO_2_/air at 37°C and imaged with a Zeiss LSM 510 confocal microscope (Thornwood, NY). Both Integrated Brightness of mitotracker and TMRM assay were measured and analyzed by Keyence BZ-X Analyzed software.

### Extracellular flux assays

Bioenergetics profile of transient RARRES1 or CCP2 knockdown in MCF10A and PWR-1E cells and stable RARRES1 knockdown MCF10A cells were measured using the XF^e^96 Extracellular Flux Analyzer. 48 hours prior to analysis, MCF10A and PWR-1E cells were transfected with RARRES1 or CCP2 siRNA or scrambled siRNA. The night before the assay, the cells were seeded at an optimized cell density (MCF10A cells: 10,000 and PWR-1E cells: 20,000) in the 96-well XF^e^ plate and incubated at 37°C with 5% CO2 overnight. The day of analysis, the cells were incubated at 37°C in a CO_2_-free atmosphere incubator for 1 hour. Basal oxygen consumption rate and extracellular acidification rate were measured using XF Cell Mito Stress Test. Subsequently, oxygen consumption rate (OCR) and extracellular acidification rate (ECAR) responses were observed after separate injections of oligomycin (1 uM), carbonyl cyanide-p-trifluoromethoxyphenylhydrazone FCCP (0.5 uM for MCF10A cells or 0.25 uM for PWR-1E cells), and a combination of rotenone and antimycin A (0.5 uM) were respectively prompted in the assay. Glycolysis was analyzed, using the XF Glycolysis Stress Test kit, in the XFe96 Extracellular Flux Analyzer. ECAR was measured at baseline and after adding glucose (10 mM), oligomycin (1uM) and 2-deoxy-d-glucose (100 mM) respectively. The glycolytic Rate Assay was also run on transiently CCP2 depleted cells and control cells (transfected with scrambled siRNA). Cells were grown in media with 10 mM glucose prior to Seahorse extracellular flux assay. During the flux assay, ECAR was measured at baseline and after injecting antimycin A and rotenone (0.5 uM) and 2-deoxy-d-glucose (100 mM).

### LC-MS analysis and metabolite identification

Ultra-performance Liquid Chromatography-Time of Flight Mass Spectrometry (UPLC TOF MS) based metabolomic analyses was done on RARRES1 stable knockdown and the control empty vector MCF 10A cells. See [20] for the running, metabolite extraction, data preprocessing, statistical analyses methods and identification of metabolites. The metabolite identifications (Supplementary Table 1) were confirmed by comparing the retention time under the same chromatographic conditions and by matching the fragmentation pattern of the parent ion from the biological sample to that of the standard metabolite using tandem mass spectrometry (UPLC-TOFMS/MS).

### Statistical analysis

*In vitro* data analysis. Results are shown as the mean ± SD. Statistical significance was calculated by using Student’s *t*-test and *P* < 0.05 was accepted as significant value (***, *p* < 0.001; **, *p* < 0.01; *, *p* < 0.05). At least three biological replicates were done to confirm the results.

## SUPPLEMENTARY MATERIALS FIGURES AND TABLE



## Abbreviations

ARRES1: Retinoic Acid Receptor Responder 1
RA: Retinoic Acid
CCP2: Cytosolic Carboxypeptidase 2
AGBL2: ATP/GTP Binding Protein Like 2
VDAC1: Voltage Dependent Anion Channel 1
MMP: Mitochondrial Membrane Potential
AMPK: AMP kinase
