# Correction: Tumor suppressor RARRES1 links tubulin deglutamylation to mitochondrial metabolism and cell survival

**DOI:** 10.18632/oncotarget.28093

**Published:** 2021-10-26

**Authors:** Sara Maimouni, Mi-Hye Lee, You-Me Sung, Michael Hall, Arpita Roy, Chokri Ouaari, Yoo-Seok Hwang, Justin Spivak, Eric Glasgow, Matthew Swift, Jay Patel, Amrita Cheema, Deepak Kumar, Stephen Byers

**Affiliations:** ^1^Department of Oncology, Georgetown-Lombardi Comprehensive Cancer Center, Georgetown University, Washington, DC, USA; ^2^Department of Biochemical, Molecular and Cellular Biology, Georgetown University, Washington, DC, USA; ^3^University of the District of Columbia, Washington, DC, USA; ^4^Cancer & Developmental Biology Laboratory, National Cancer Institute-Frederick, Frederick, MD, USA; ^*^These authors have contributed equally to this article

**This article has been corrected:** During reorganization of Figure 1, the authors intended to show HFK cells, not PWRE cells, in panel C. Unfortunately, they kept the labeling but inserted the PWRE blot instead of the HFK blot. The proper Figure 1 is shown below. The authors declare that these corrections do not change the results or conclusions of this paper.

Original article: Oncotarget. 2019; 10:1606–1624. 1606-1624. https://doi.org/10.18632/oncotarget.26600

**Figure 1:**
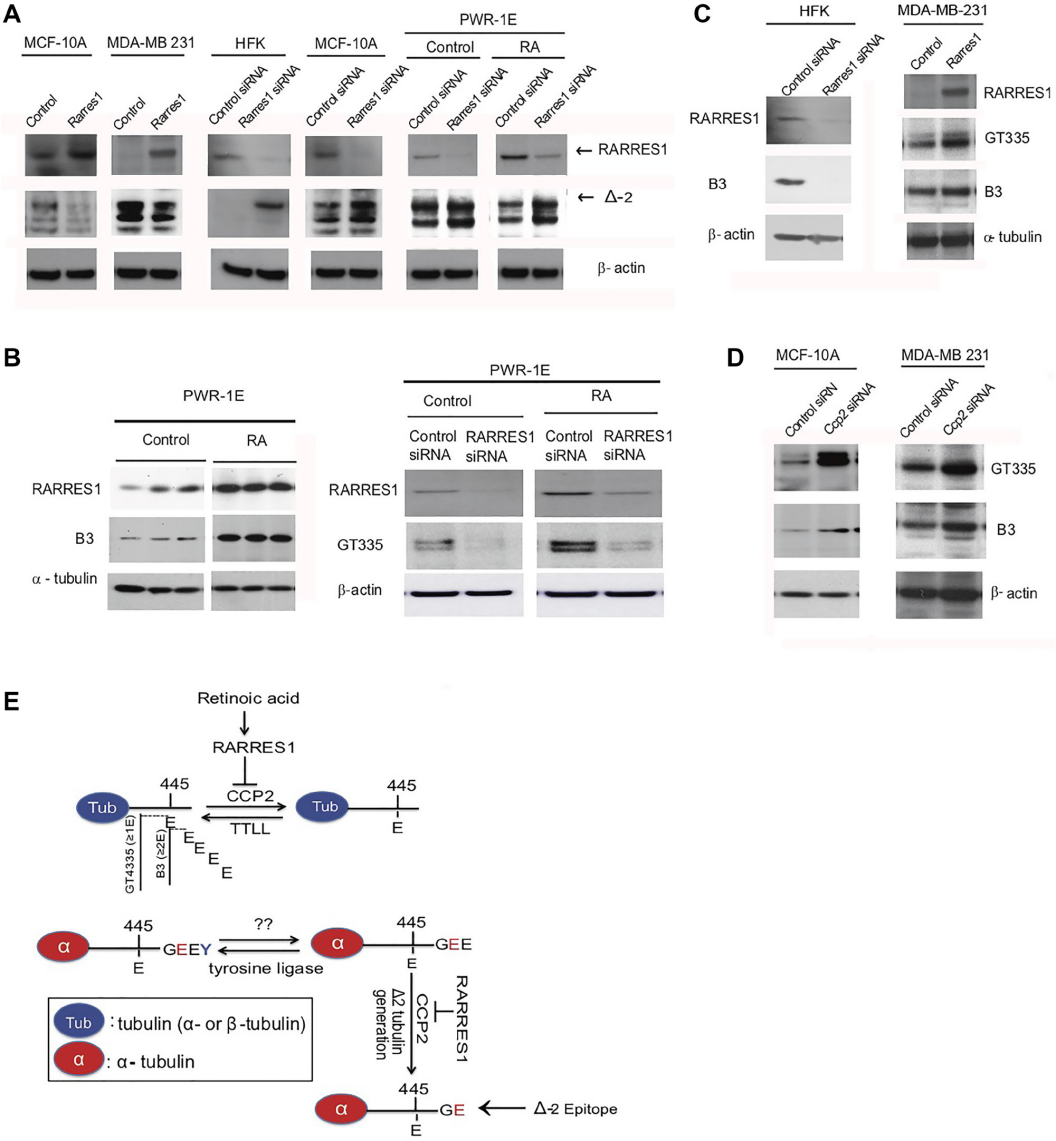
RARRES1, CCP2 and Retinoic acid Regulate Tubulin Glutamylation. (**A**) Δ-2 tubulin levels are regulated by RARRES1 in MDA-10A, MDA-231, HFK and PWR-1E cells. (**B**) RARRES1 and polyglutamylated tubulin is increased by retinoic acid (10^−7^M all-trans-RA for 24 hours). Western blots in PWR-1E cells three biological replicates were run for each experimental condition (Vehicle (control) vs. RA treatment). RARRES1 siRNA reversed its effect on polyglutamylation. The B3 antibody, which detects tubulin side-chains containing two or more glutamates, was used. (**C**) Immunoblot for B3 was also assessed in HFK cells with RARRES1 or control knockdowns. Immunoblots for GT335, which detects one or more glutamates attached to the side chain, and B3 were done to assess the effects of RARRES1 exogenous expression in MDA-MB-231 cells, in which RARRES1 is slienced. (**D**) Immunoblot of CCP2 and polyglutamylated tubulin in CCP2 knockdown MCF10A and MDA-MB-231 cells. (**E**) Schematic illustrating the effects of RA and RARRES1 in CCP2 regulation of tubulin deglutamylation. Epitopes of GT335, B3 and Δ-2 antibodies are also shown.

