# Supplementary material for: Tumor suppressor RARRES1 links tubulin deglutamylation to mitochondrial metabolism and cell survival
Source: Oncotarget. 2019 Feb 26;10(17):1606–24. doi: 10.18632/oncotarget.26600 (PMC6422194; doi:10.18632/oncotarget.26600)
Supplement: Supplementary file 1 [file oncotarget-10-1606-s001.pdf]

Tumor suppressor RARRES1 links tubulin deglutamylation to mitochondrial metabolism and cell survival

SUPPLEMENTARY MATERIALS

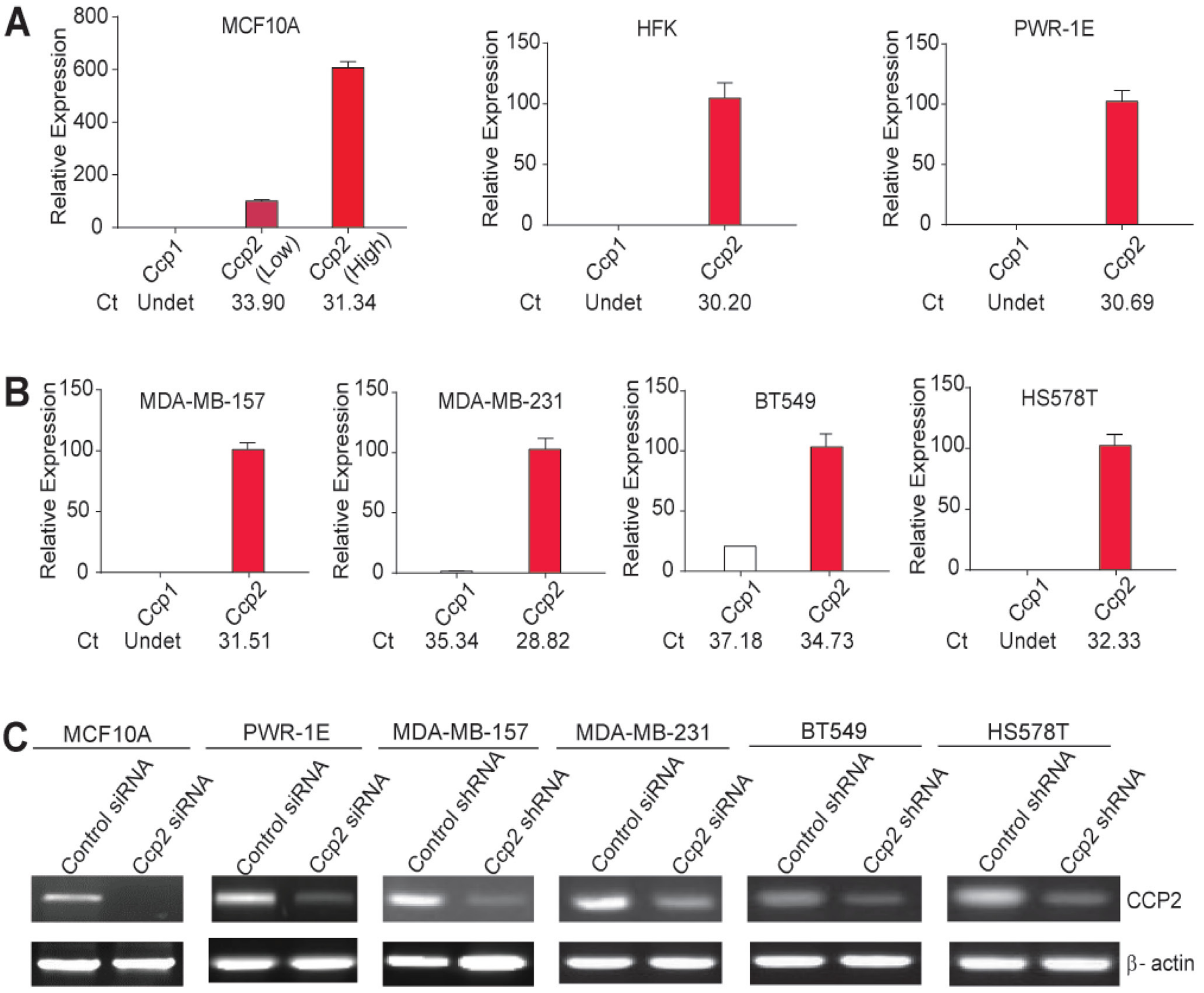

Supplementary Figure 1: CCP2 expression in tumorigenic and non-tumorigenic cell lines. A. Relative mRNA expression of CCP1 and CCP2 in non-tumorigenic cell lines, MCF10A, HFK, and PRWR-1E. CCP2 expression is regulated by cell density in MCF10A cells. **B.** Relative mRNA expression of CCP2 and CCP2 in tumorigenic cell lines, MDA-MB-157, MDA-MB-231, BT547, and HS578T. **C.** CCP2 knockdown in several cell lines. These results were confirmed by qPCR (data not shown). Refer to Supplementary Figure 17 for full-length gels.

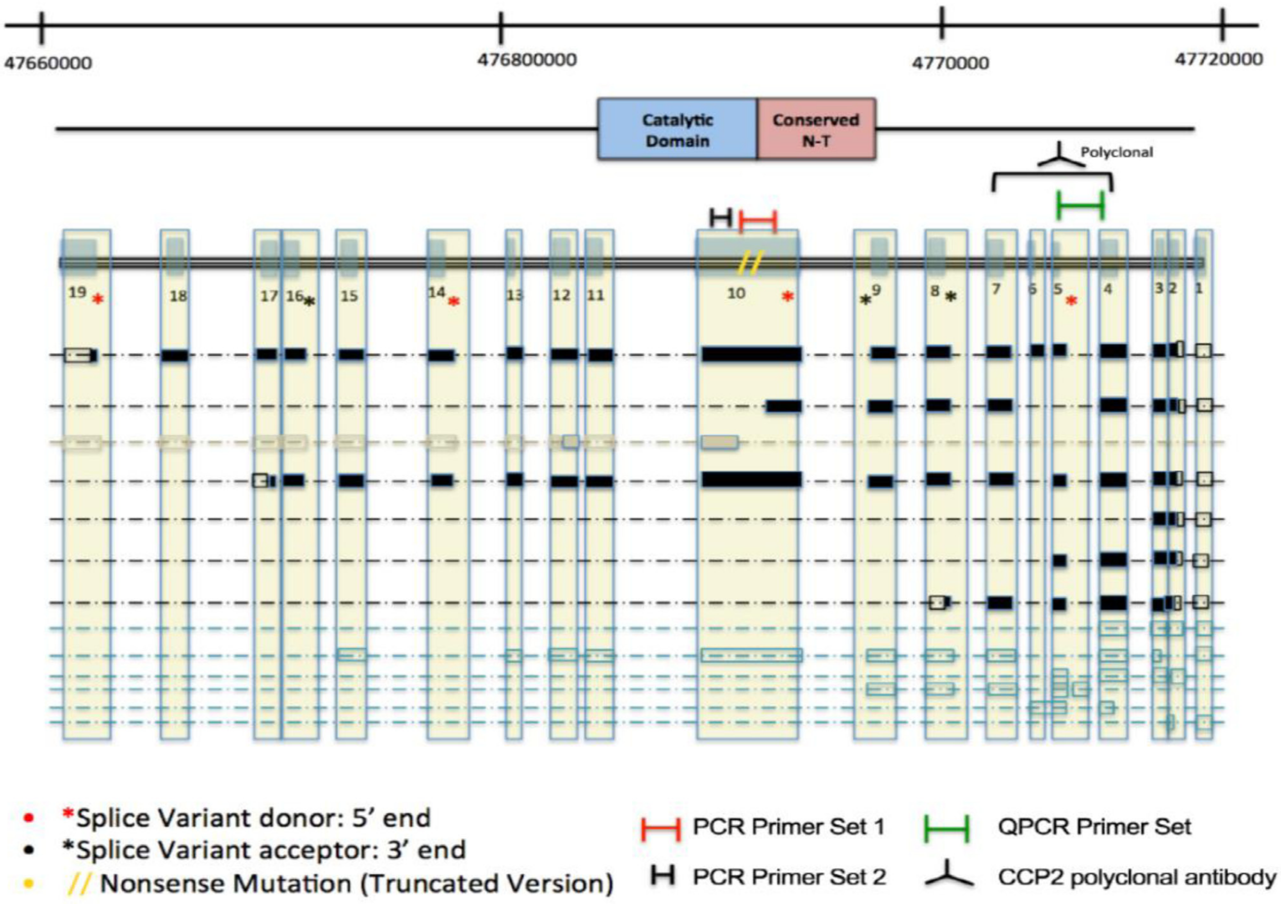

Supplementary Figure 2: CCP2 splice variants and primer profiling the CCP2 gene contains over 13 variants six of which are translated (Harrow et al., 2006; ENSEMBL). The red and black asterisks are splice variant regions predicted by ENSEMBL, where red is the splice donor and the black signifies the splice acceptor. Our primer sets, we were able to detect four of the known translated variants. This includes the full length variant and the catalytically viable version with a truncated c-terminus (exons 19,18,and 17), three processed transcripts and one nonsense-mediated decay transcript. Our polyclonal antibody detects five out of the six translated splice variants.

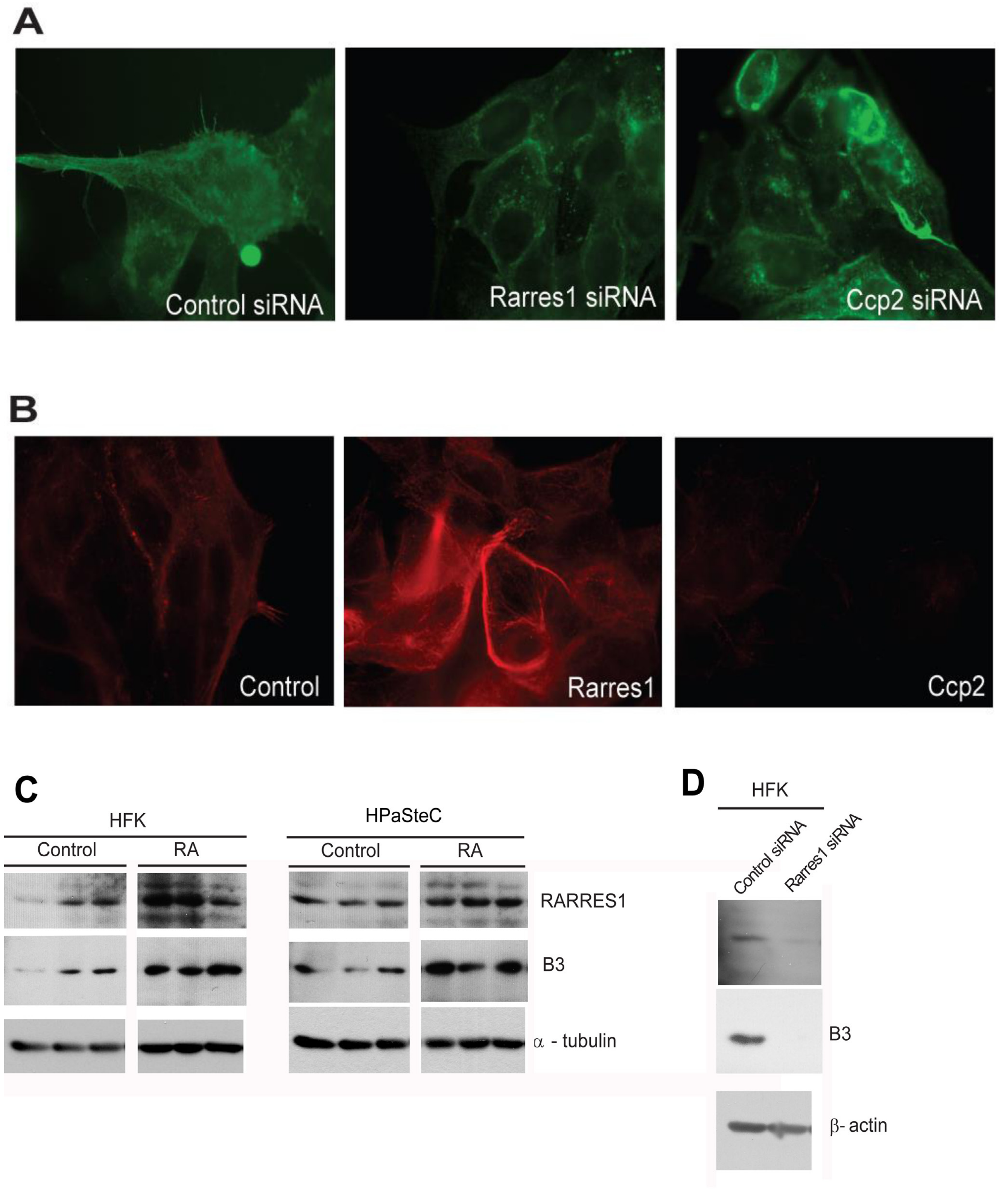

Supplementary Figure 3: Effects of RARRES1 and CCP2 on polyglutamylation of tubulin. A. Immunocytochemistry and immunoblot of polyglutamylated tubulin (antibody B3) in RARRES1 depleted **B.**, and expressing PWRE-1 cells. **C.** Retinoic acid and its effects on polyglutamylation, 2 glutamates (biglutamylation) or more attached to the side of chain of tubulin (detected by B3 antibody). **D.** Biglutamylation or polyglutamylation, detected by B3 antibody, of tubulin side chains in RARRES1-depleted HFK cells vs. control-siRNA transfected HFK cells.

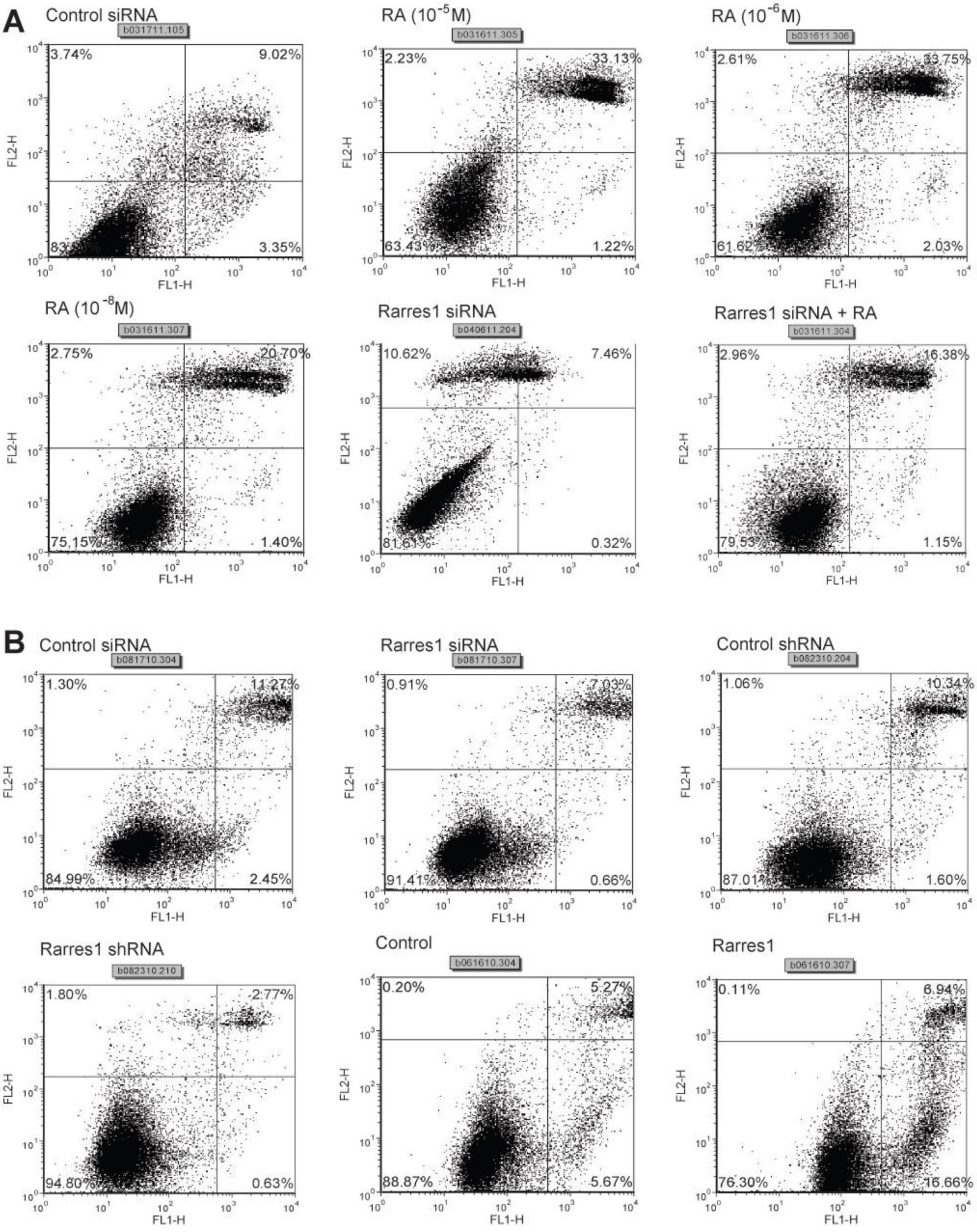

Supplementary Figure 4: FACS analysis of annexin stained cells. A. RARRES1 knockdown MCF10A without or with retinoic acid in dose dependant manner. **B.** MCF10A controls, RARRES1 transient knockdown, and RARRES1 stable knockdown, and RARRES1 overexpression. Samples were stained with fluorescein-labeled Annexin V and propidium iodide (Sigma) and analyzed by flow cytometry to measure apoptosis. Experiments were repeated three times with consistent and repeatable results.

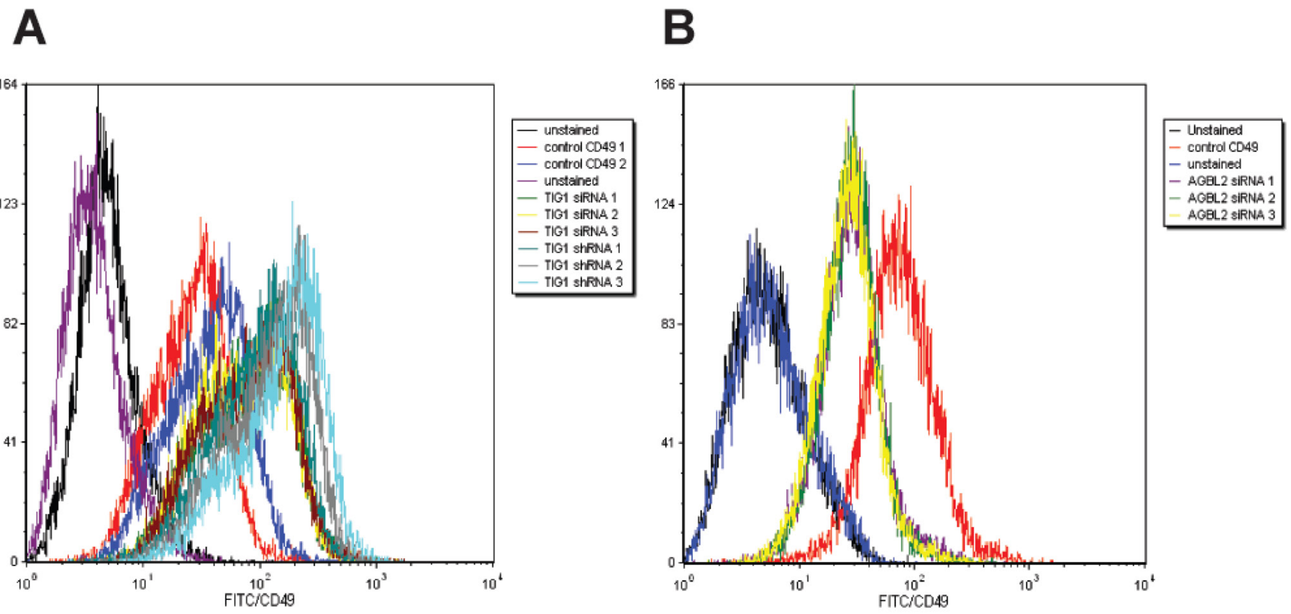

Supplementary Figure 5: RARRES1 and CCP2 reciprocally regulate stem cell marker CD49f. A. Results for the flow cytometry for CD49f demonstrates increased cell surface CD49f following RARRES1 knockdown by siRNA and culture of MCF10A cells. **B.** CCP2 knockdown reduces CD49f cell surface expression.

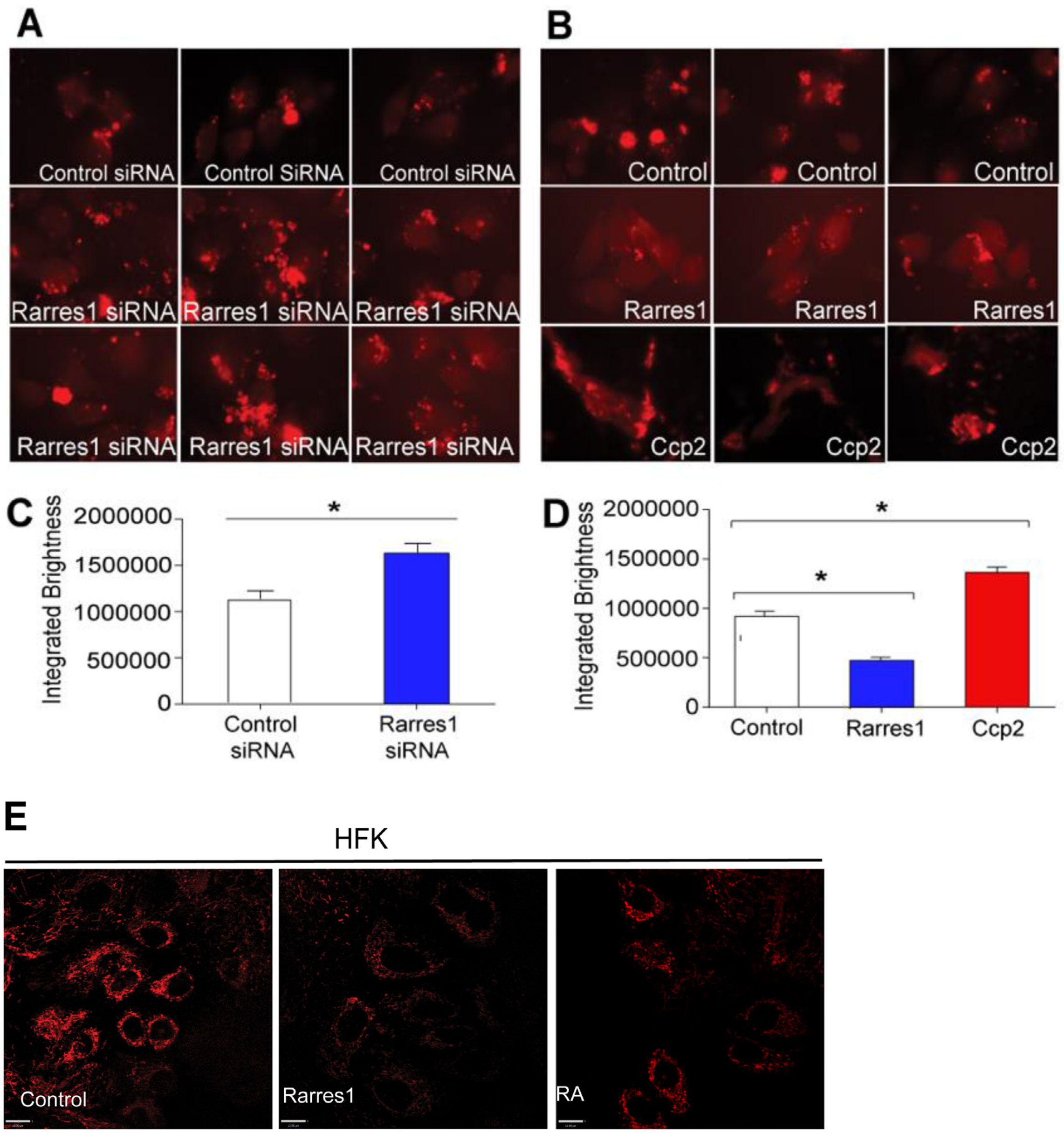

Supplementary Figure 6: Mitotracker staining and mitochondrial respiration after simultaneous depletion of CCP2 and RARRES1. A. and **B.** Mitotracker staining in PWR-1E control, RARRES1 and CCP2 manipulated cells. **C.** and **D.** Integrated brightness of mitotracker measured by Keyence analysis software. **E.** MMP measurement by TMRM in HFK cells after RARRES1 or empty vector exogenous expression and RA treatment or vehicle treatment. TMRM was run after 24 hours of treatment or transfection

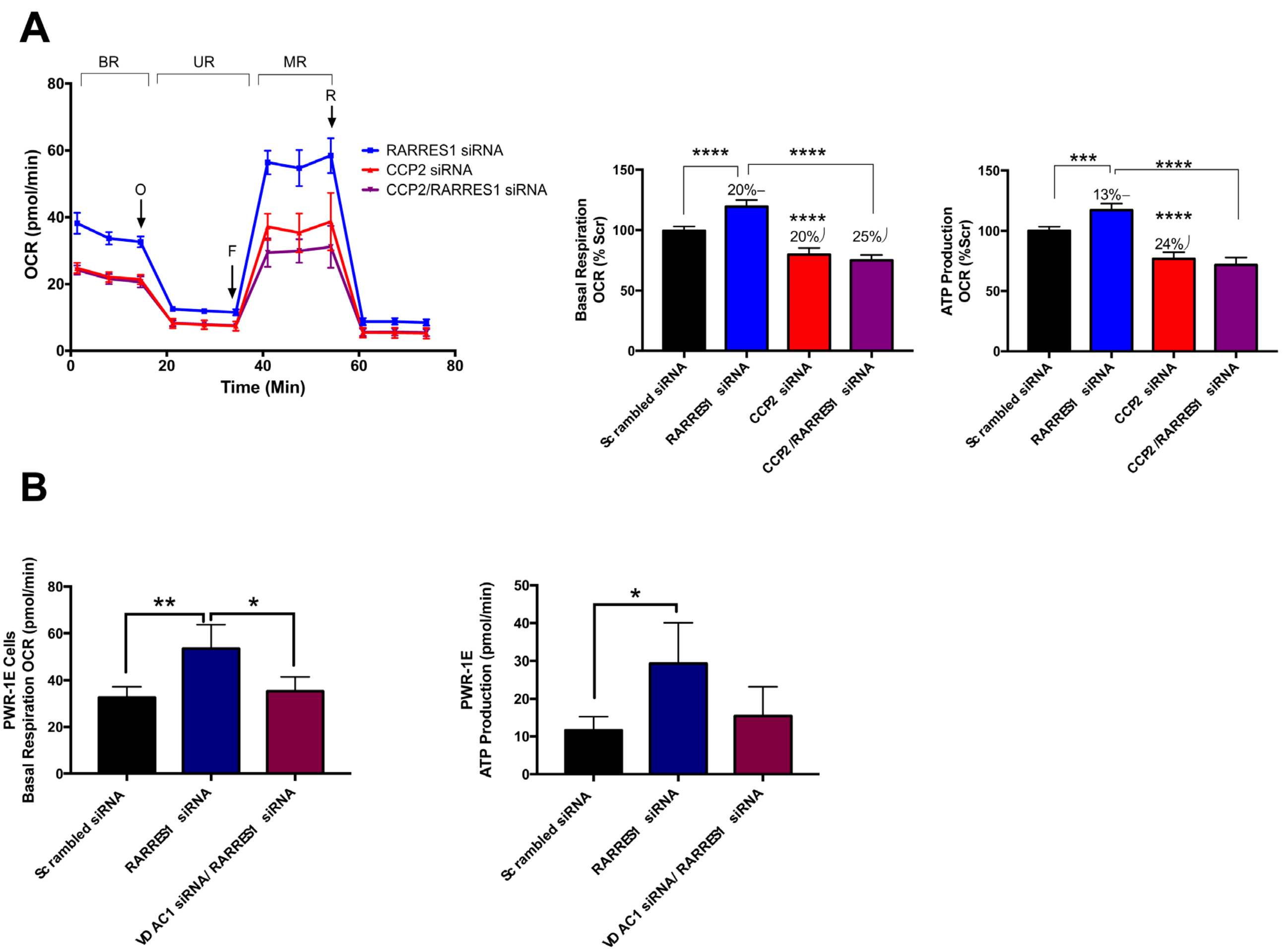

Supplementary Figure 7: CCP2/RARRES1 double knockdown and VDAC1/RARRES1 double knockdown and their effects on mitochondrial respiration. **A.** OCR activity after simultaneous transient knockdown of CCP2 and RARRES1 in MCF 10A cells. **B.** PWR-1E cells were transfected with scrambled siRNA, RARRES1 siRNA or VDAC1 and RARRES1 siRNAs. The OCR after FCCP injection and oligomycin injection were quantified.

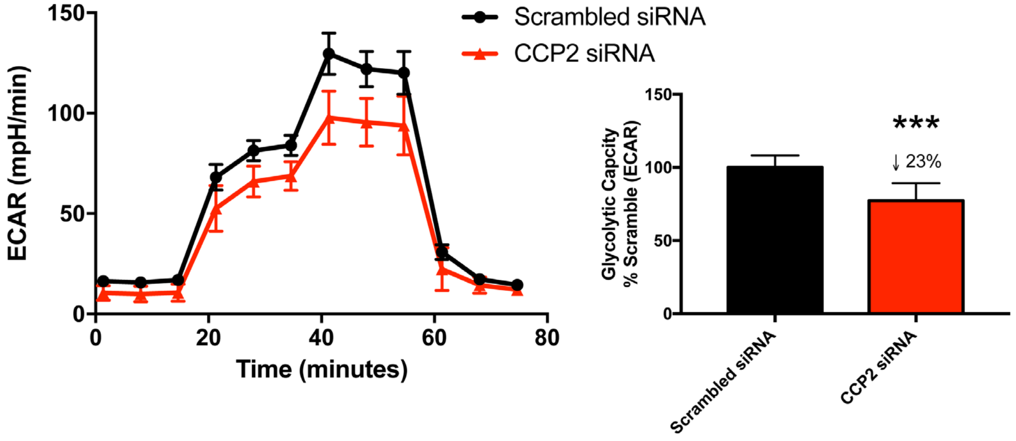

Supplementary Figure 8: Glycolytic capacity of CCP2-depleted PWR-1E cells. PWR-1E cells were transfected with scrambled siRNA or CCP2 siRNA were starved of glucose 1 hour prior to the assay and a combination of glucose, oligomycin 2- deoxy-d-glucose were injected. Glycolytic capacity is measured by calculating the difference between ECAR after oligomycin injection and 2 deoxy-d-glucose injection.

A

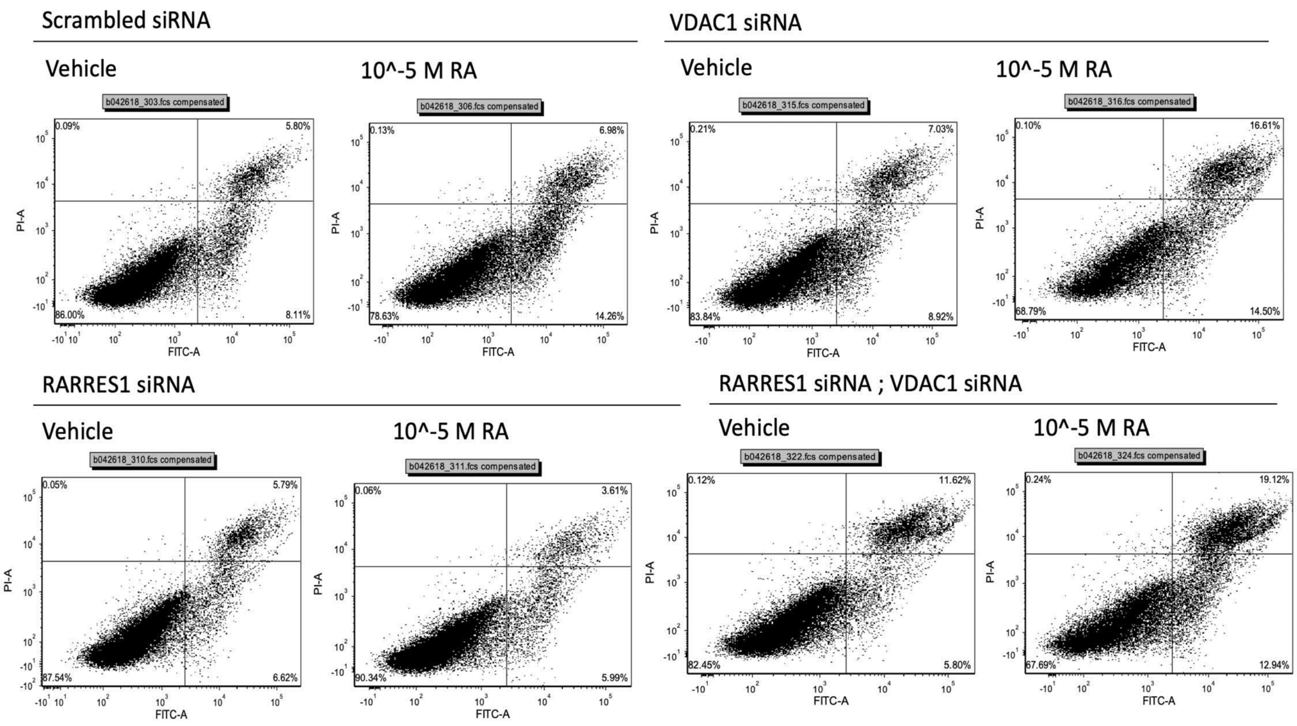

B

Scrambled siRNA 2 μM Itraconazole RARRES1 siRNA 2 μM Itraconazole

Supplementary Figure 9: FACS analysis of annexin stained cells. A. MCF10A controls, RARRES1 transient knockdown, VDAC1 siRNA and RARRES1 siRNA in combination with VDAC1 siRNA were treated with 10^-5 M retinoic acid or vehicle. Samples were stained with fluorescein-labeled Annexin V and propidium iodide (Sigma) and analyzed by flow cytometry to measure apoptosis. **B.** Scrambled siRNA and RARRES1 siRNA were treated with itraconazole. Cells were stained with fluorescein-labeled Annexin V and propidium iodide and analyzed by flow cytometry to measure apopoptosis.

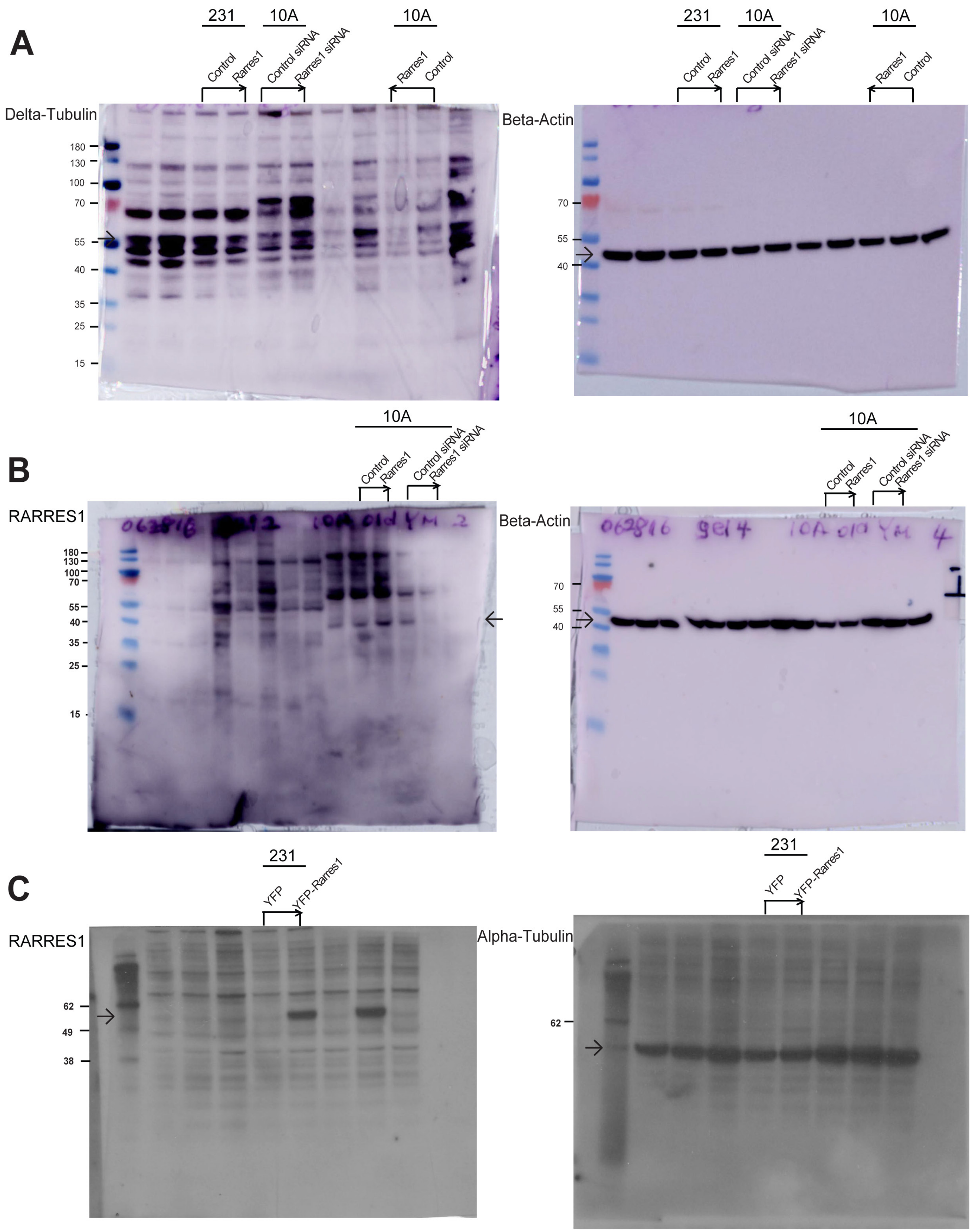
A
231
10A
10A
Control
Rarres1
Control siRNA
Rarres1 siRNA
Rarres1
Control
Delta-Tubulin
180
130
100
70
55
40
35
25
15
231
10A
10A
Control
Rarres1
Control siRNA
Rarres1 siRNA
Rarres1
Control
Beta-Actin
70
55
40
B
10A
Control
Rarres1
Control siRNA
Rarres1 siRNA
RARRES1
180
130
100
70
55
40
35
25
15
10A
Control
Rarres1
Control siRNA
Rarres1 siRNA
Beta-Actin
70
55
40
C
231
YFP
YFP-Rarres1
RARRES1
62
49
38
231
YFP
YFP-Rarres1
Alpha-Tubulin
62

D

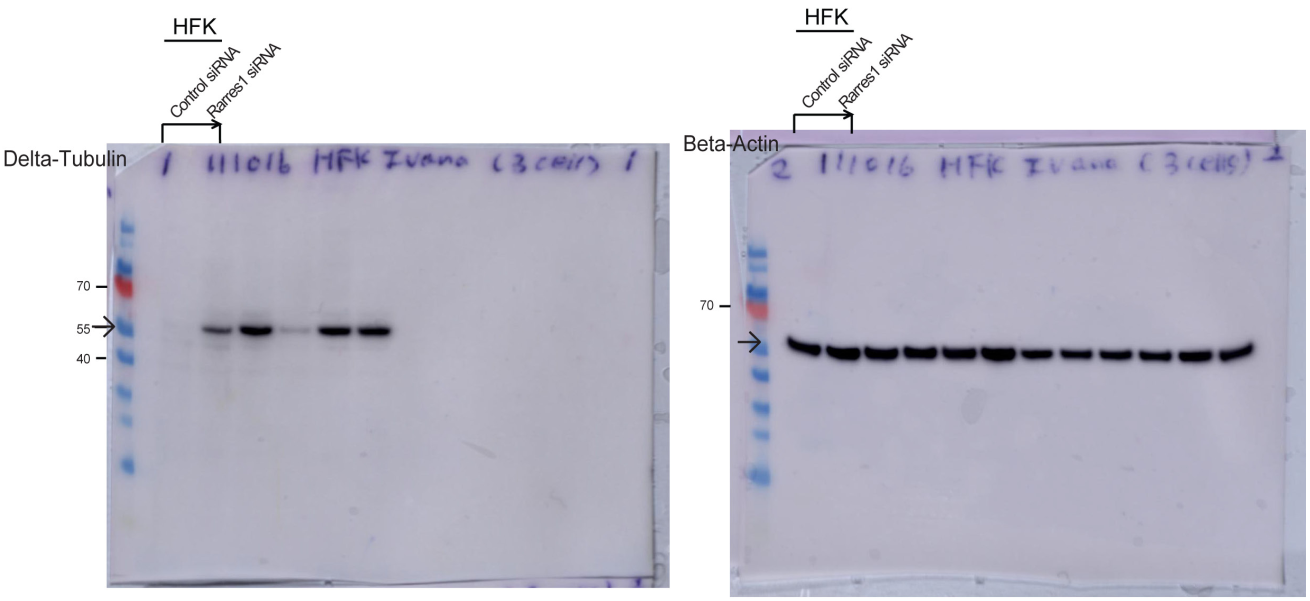
HFK
Control siRNA
Rarres1 siRNA
Delta-Tubulin
70
55
40
HFK
Control siRNA
Rarres1 siRNA
Beta-Actin
70

E

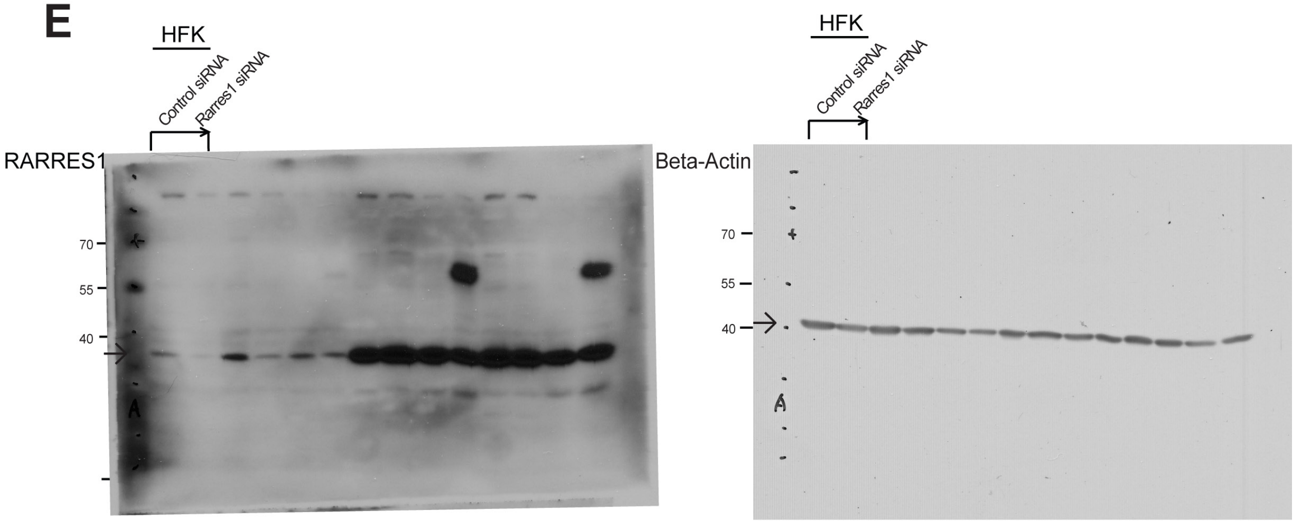
HFK
Control siRNA
Rarres1 siRNA
RARRES1
70
55
40
HFK
Control siRNA
Rarres1 siRNA
Beta-Actin
70
55
40

F

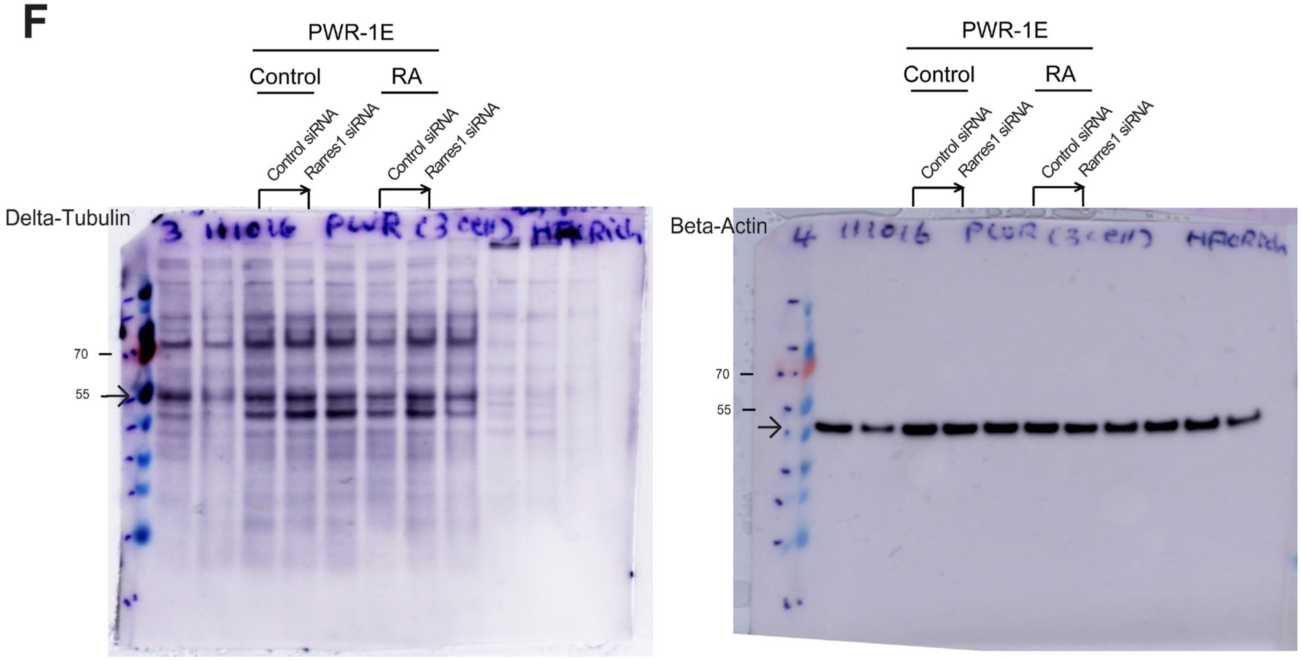
PWR-1E
Control
RA
Control siRNA
Rarres1 siRNA
Control siRNA
Rarres1 siRNA
Delta-Tubulin
70
55
PWR-1E
Control
RA
Control siRNA
Rarres1 siRNA
Control siRNA
Rarres1 siRNA
Beta-Actin
70
55

G

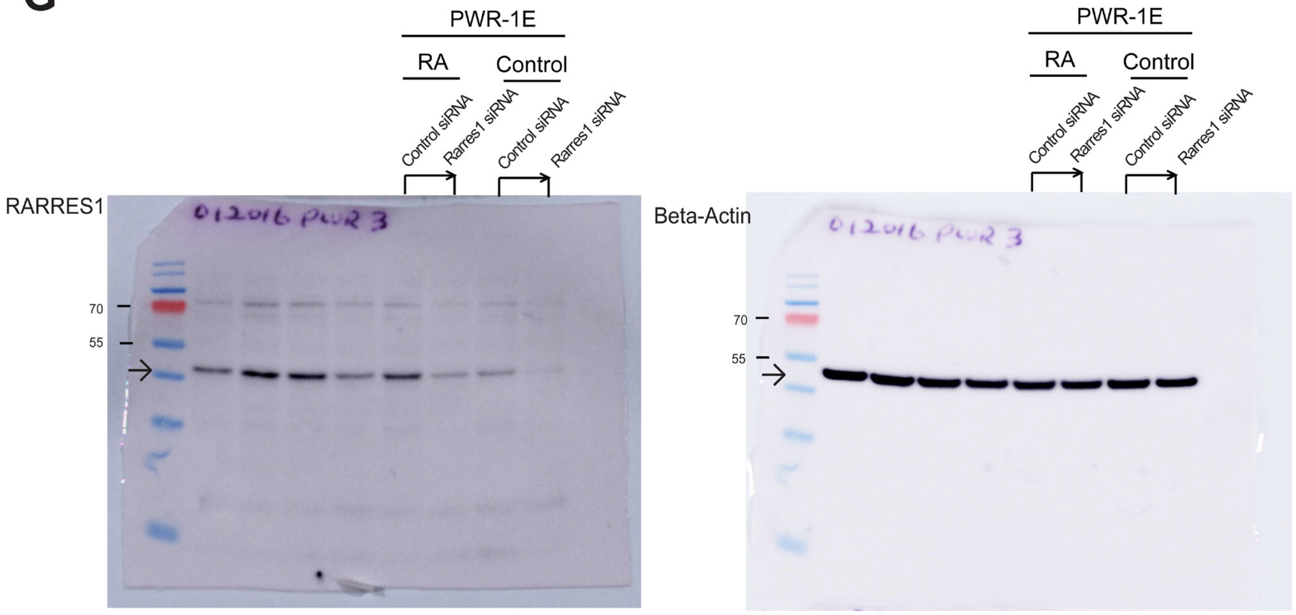
PWR-1E
RA
Control
Control siRNA
Rarres1 siRNA
Control siRNA
Rarres1 siRNA
RARRES1
70
55
PWR-1E
RA
Control
Control siRNA
Rarres1 siRNA
Control siRNA
Rarres1 siRNA
Beta-Actin
70
55

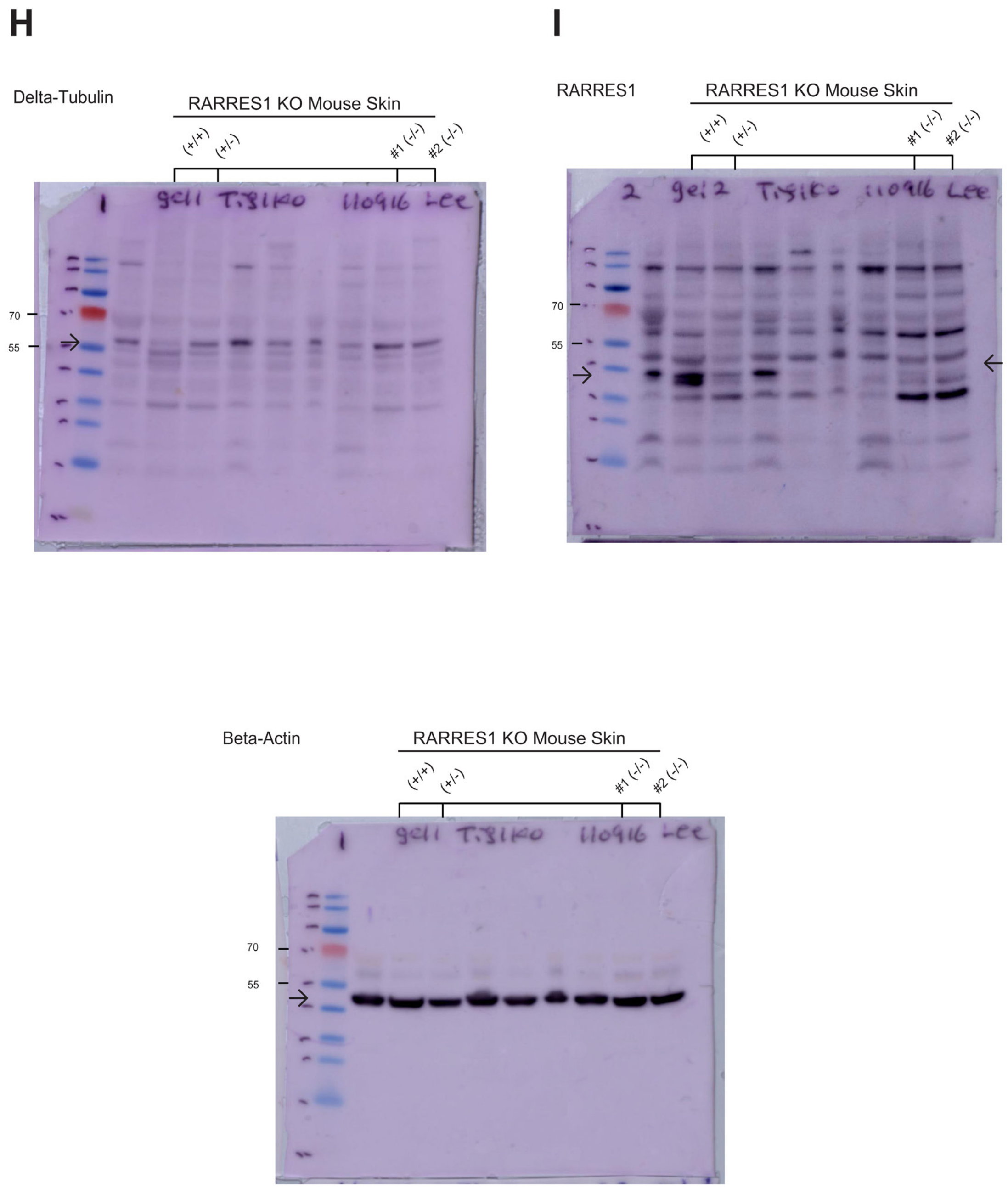

Supplementary Figure 10: Full-length immunoblots for Figure 1A: Delta 2 tubulin levels are regulated by RARRES1 in MDA-10A, MDA-231, HFK, PWR-1E cells and in RARRES1-/- mouse skin. **A.** Full- length western blots of RARRES1 overexpression and /or RARRES1- transient knockdown in MDA-MB-231 and MCF 10A cells are represented. The left blot is probed for delta 2 tubulin while the blot on the right is probed for beta actin. The lanes with no bracket/labeling are irrelevant to this study. **B.** Full-length blots of RARES1- overexpression and – transient knockdown in MCF 10A cells are represented. The left blot is probed for RARRES1 while the blot on the right is probed for beta actin as an internal control. The lanes with no bracket/labeling are irrelevant to this study. **C.** Full-length blot of RARRES1-YFP overexpression in MDA-MB-231 is represented in left side and its expression was validated as the expected band ~ 60Kda. Alpha-tubulin was selected as a loading control on right side. **D.**, **E.** Full-length western blots of RARRES1 transient knockdown from human neonatal foreskin primary epidermal keratinocyte (HFK) are pictured. The left blots are probed for delta 2 tubulin and RARRES1 while the blots on the right are probed for beta actin. The lanes that are relevant to this study are highlighted in black borders. Control cells with scrambled siRNA and RARRES1 siRNA transfected HFK cells were assessed. **F.**, **G.** Full length Immunoblots of delta 2 tubulin and RARRES1 after RARRES1 manipulation with or without retinoic acid in PWR-1E cells. The left blots are probed for delta 2 tubulin and RARRES1 while the blots on the right are probed for beta actin. Arrows point the locations of antibody-specific bands used in the figures. **H.**, **I.** Full-length immunoblots of delta 2 tubulin and RARRES1 protein level in mouse skin tissues of homozygous, heterozygous and wildtype RARRES1 knockout mice. Arrows point at the locations of bands used in the figures. The upper-side blots are probed for delta 2 tubulin and RARRES1 while the blot on the bottom side is probed for beta actin.

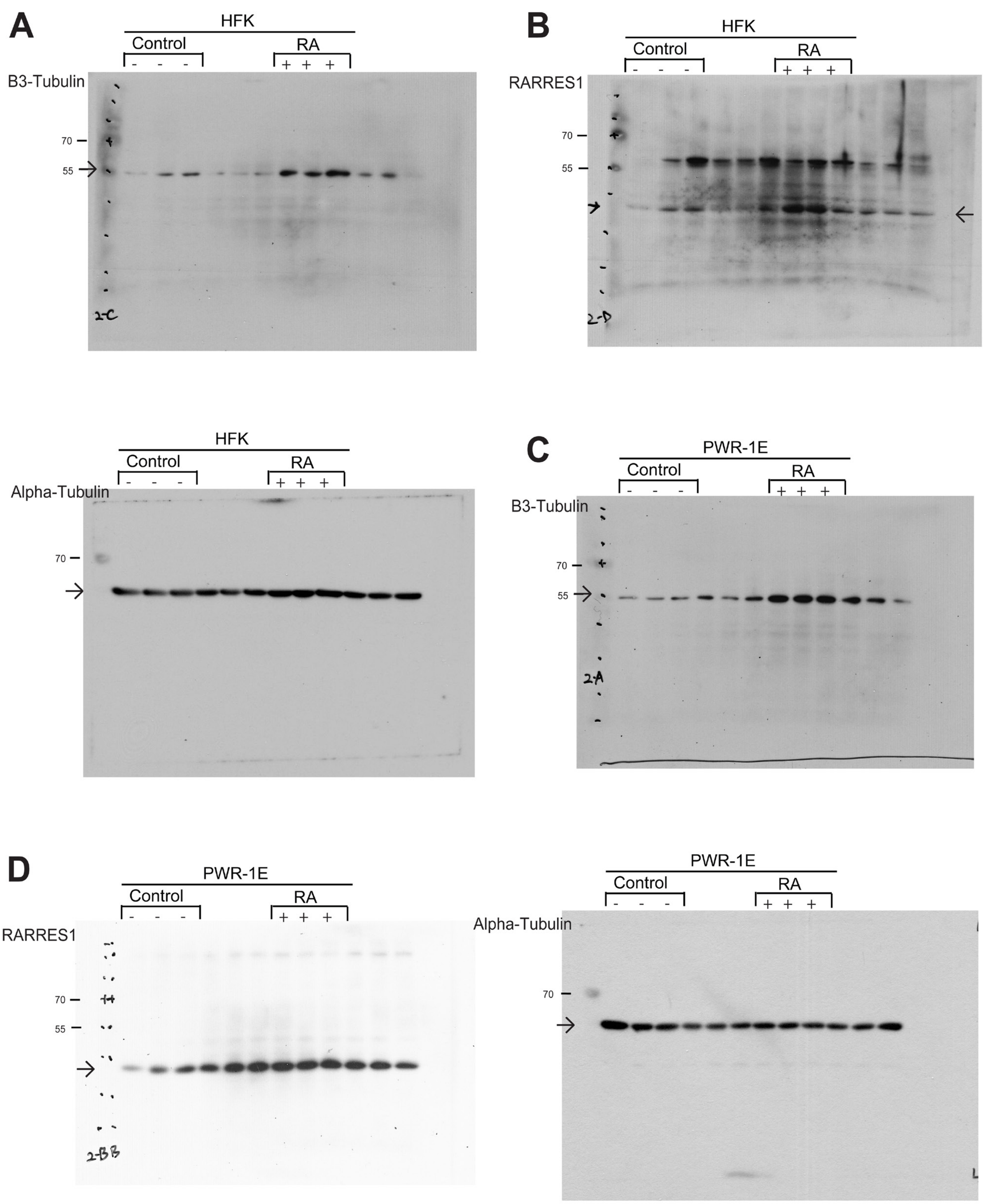
A
HFK
Control
RA
B3-Tubulin
70
55
B
HFK
Control
RA
RARRES1
70
55
HFK
Control
RA
Alpha-Tubulin
70
C
PWR-1E
Control
RA
B3-Tubulin
70
55
D
PWR-1E
Control
RA
RARRES1
70
55
PWR-1E
Control
RA
Alpha-Tubulin
70

E

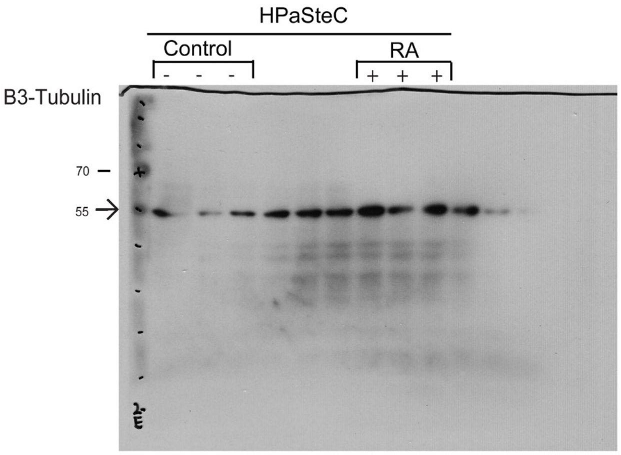

F

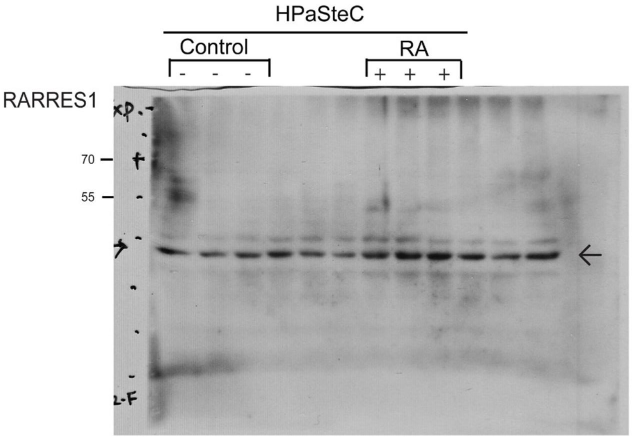

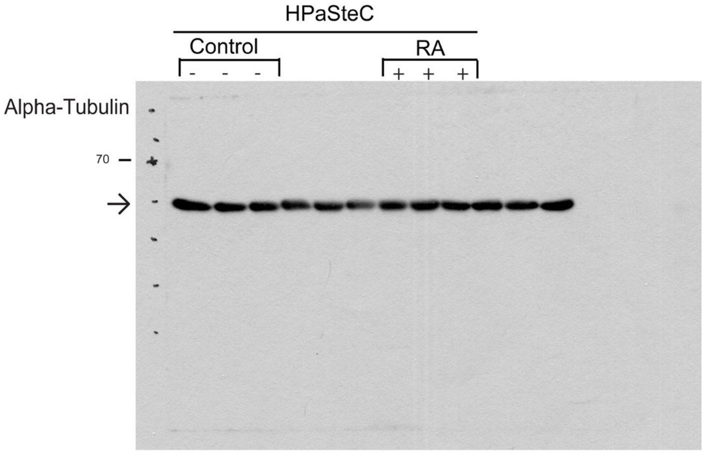

Supplementary Figure 11: Full-length immunoblots for Figure 1B: RARRES1 and B3 polyglutamylated tubulin is increased by retinoic acid (10^{-7}M all-trans-RA). A., **B.** Full-length western blots for B3- polyglutamylated tubulin and RARRES1 in HFK (human foreskin epidermal Keratinocytes) with/without the treatment of retinoic acid are presented in top side. Alpha-tubulin was selected as a loading control in middle- left side on this page. **C.**, **D.** Full-length western blots for B3- polyglutamylated tubulin and RARRES1 in PWR-1E cells with/ without the treatment of retinoic acid are presented in middle-right side and bottom-left side of this page, respectively. Alpha- tubulin was selected as a loading control in the bottom-right side of this page. **E.**, **F.** Full-length western blots for B3-polyglutamylated tubulin and RARRES1 in HPaSteC (Human Pancreatic Stellate Cell) cells with/ without the treatment of retinoic acid are presented in top and middle side on the next page, respectively. Alpha-tubulin was selected as a loading control. The lanes that are relevant to this study are highlighted in black borders. Arrows point the locations of antibody- specific bands used in the figures.

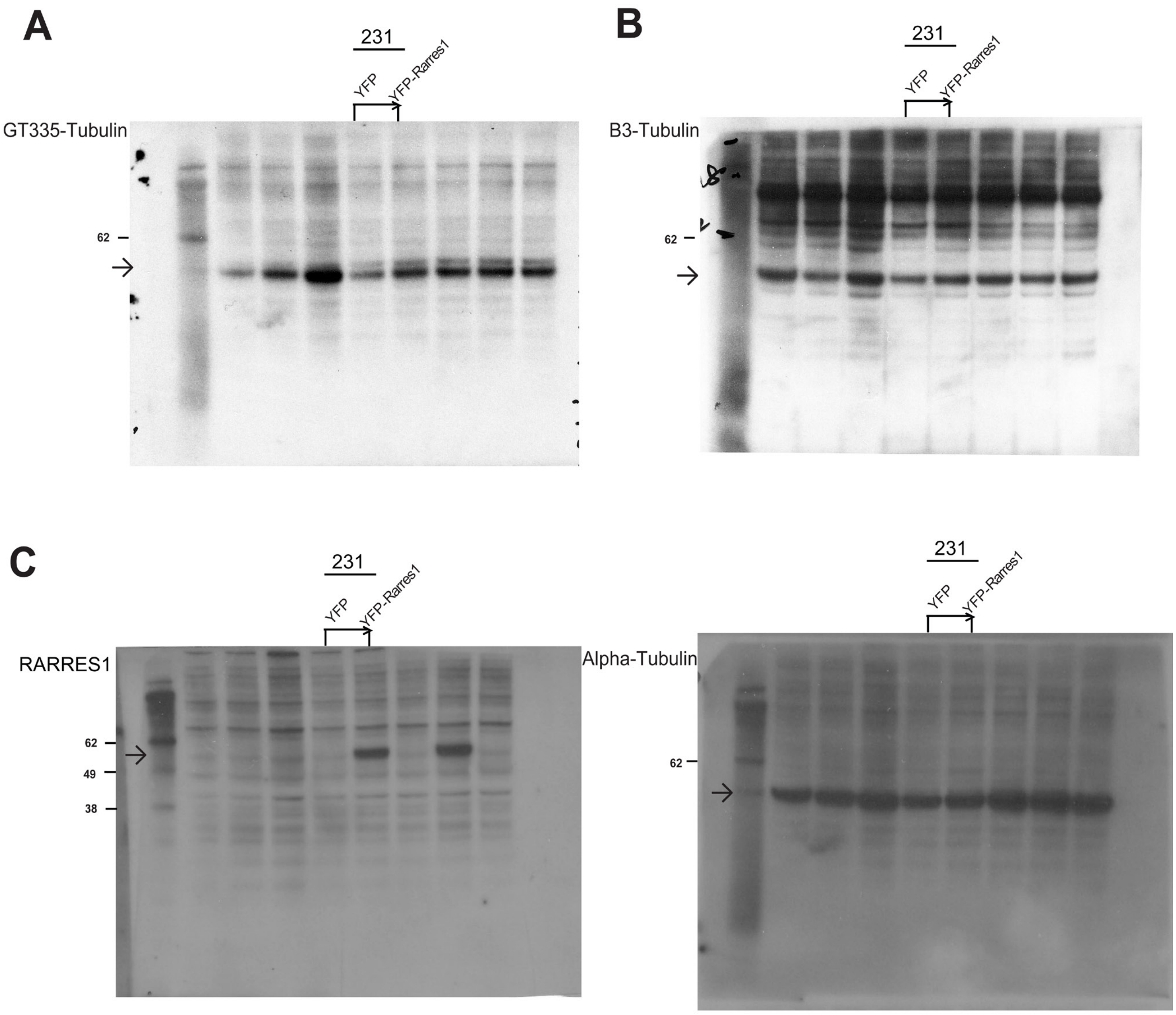

Supplementary Figure 12: Full-length immunoblots for Figure 1C: RARRES1-YFP overexpression in MDA- MB-231 is represented. **A.**, **B.** The upper blots are probed for GT335- and B3- polyglutamylated tubulin in left side and right side, respectively while the left-bottom blot is probed for RARRES1. The expressed amount of polyglutamylated tubulin and RARRES1 validated as the expected band ~55Kda and ~ 60Kda, respectively. **C.** Alpha-tubulin was selected as a loading control on bottom-right side.

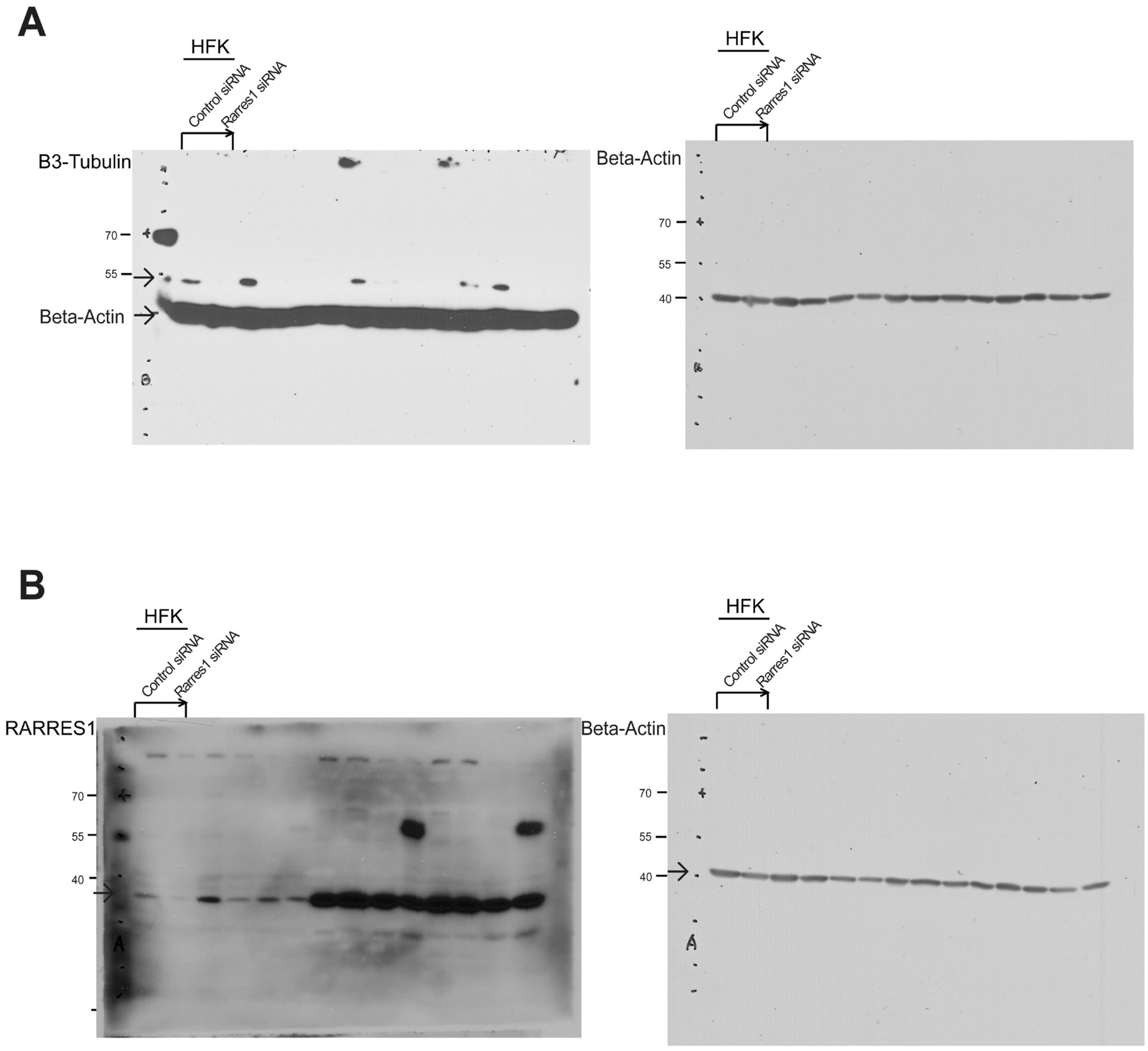
A
HFK
Control siRNA
Rarres1 siRNA
B3-Tubulin
70
55
Beta-Actin
HFK
Control siRNA
Rarres1 siRNA
Beta-Actin
70
55
40
B
HFK
Control siRNA
Rarres1 siRNA
RARRES1
70
55
40
HFK
Control siRNA
Rarres1 siRNA
Beta-Actin
70
55
40

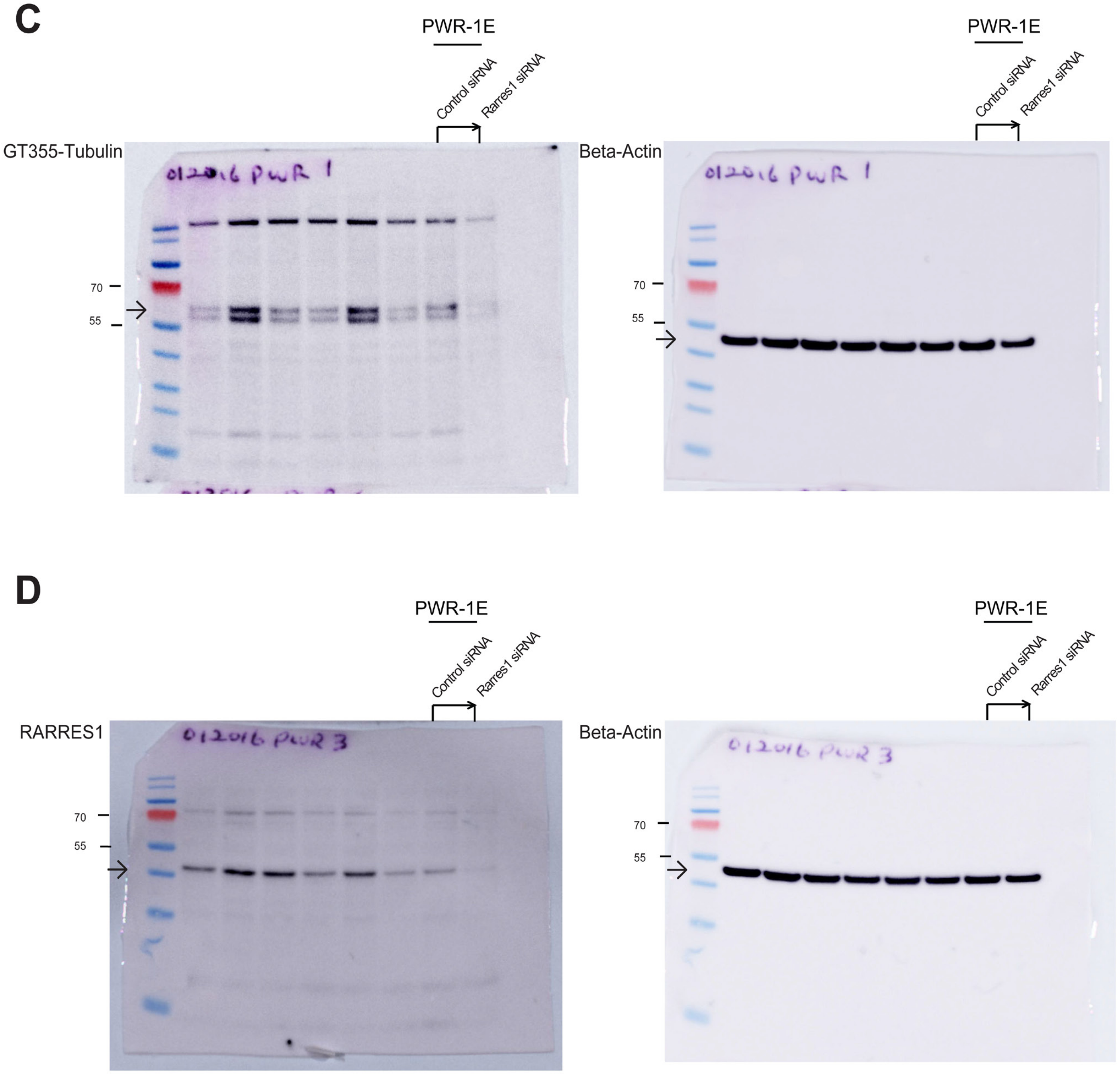

Supplementary Figure 13: Full-length immunoblots for Figure 1D: Immunoblot for RARRES1, B3 and GT335 polyglutamylated tubulin in HFK (A, B) and PWR-1E cells (C, D) in which RARRES1 was depleted. **A.**, **B.** Full-length western blots of RARRES1 transient knockdown from human neonatal foreskin primary epidermal keratinocyte (HFK) are pictured. The left blots are probed for B3 polyglutamylated tubulin and RARRES1 while the blots on the right are probed for beta actin. Control cells with scrambled siRNA and RARRES1 siRNA transfected HFK cells were assessed. Supplementary Figure 9D corresponds to Figure 1D. **C.**, **D.** Full-length western blots of RARRES1 transient knockdown from PWR-1E cells are pictured. The left blots are probed for GT-335 polyglutamylated tubulin and RARRES1 while the blots on the right are probed for beta actin. The lanes that are relevant to this study are highlighted in black borders. Control cells with scrambled siRNA and RARRES1 siRNA transfected PWR-1E cells were assessed.

A

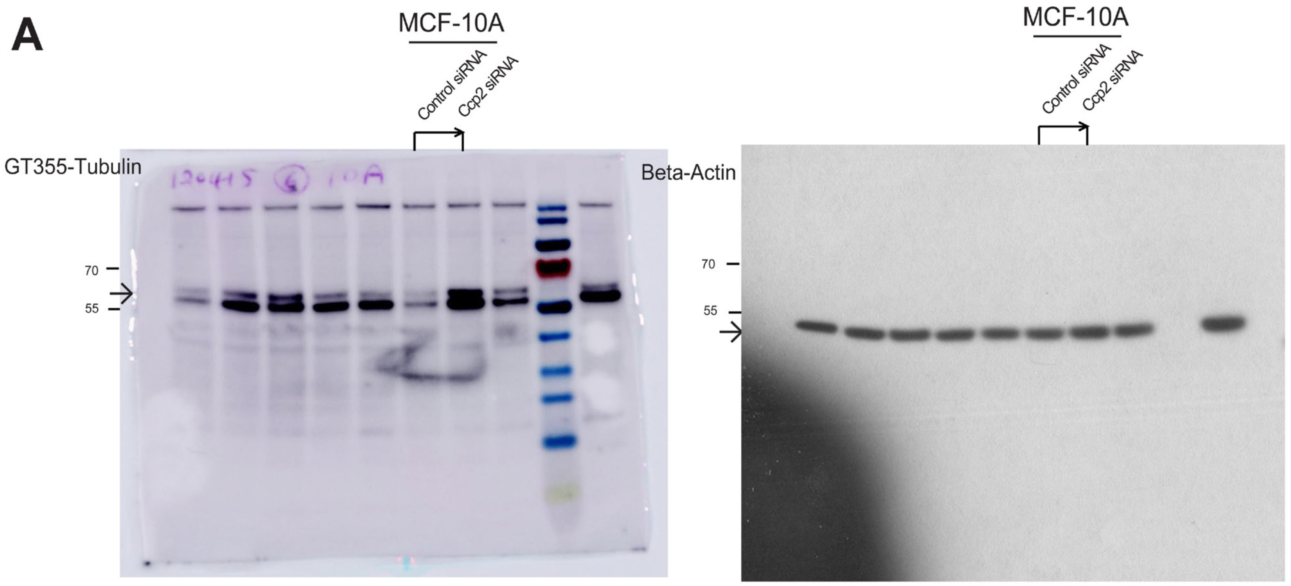
MCF-10A
Control siRNA
Ccp2 siRNA
GT355-Tubulin
70
55
MCF-10A
Control siRNA
Ccp2 siRNA
Beta-Actin
70
55

B

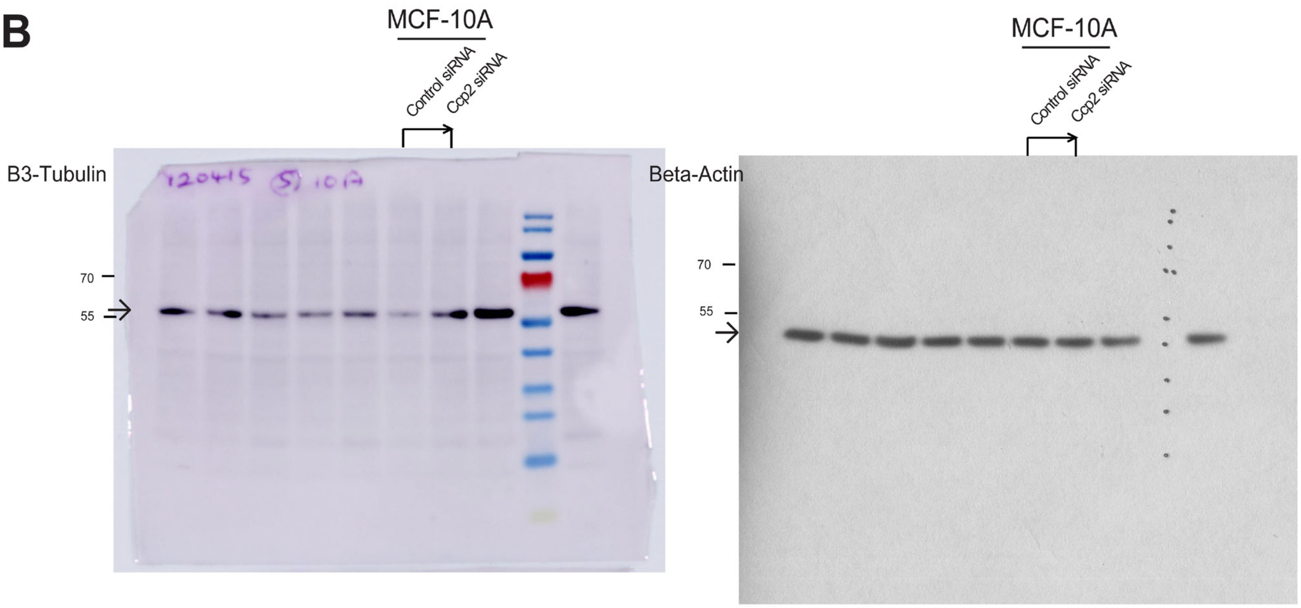
MCF-10A
Control siRNA
Ccp2 siRNA
B3-Tubulin
70
55
MCF-10A
Control siRNA
Ccp2 siRNA
Beta-Actin
70
55

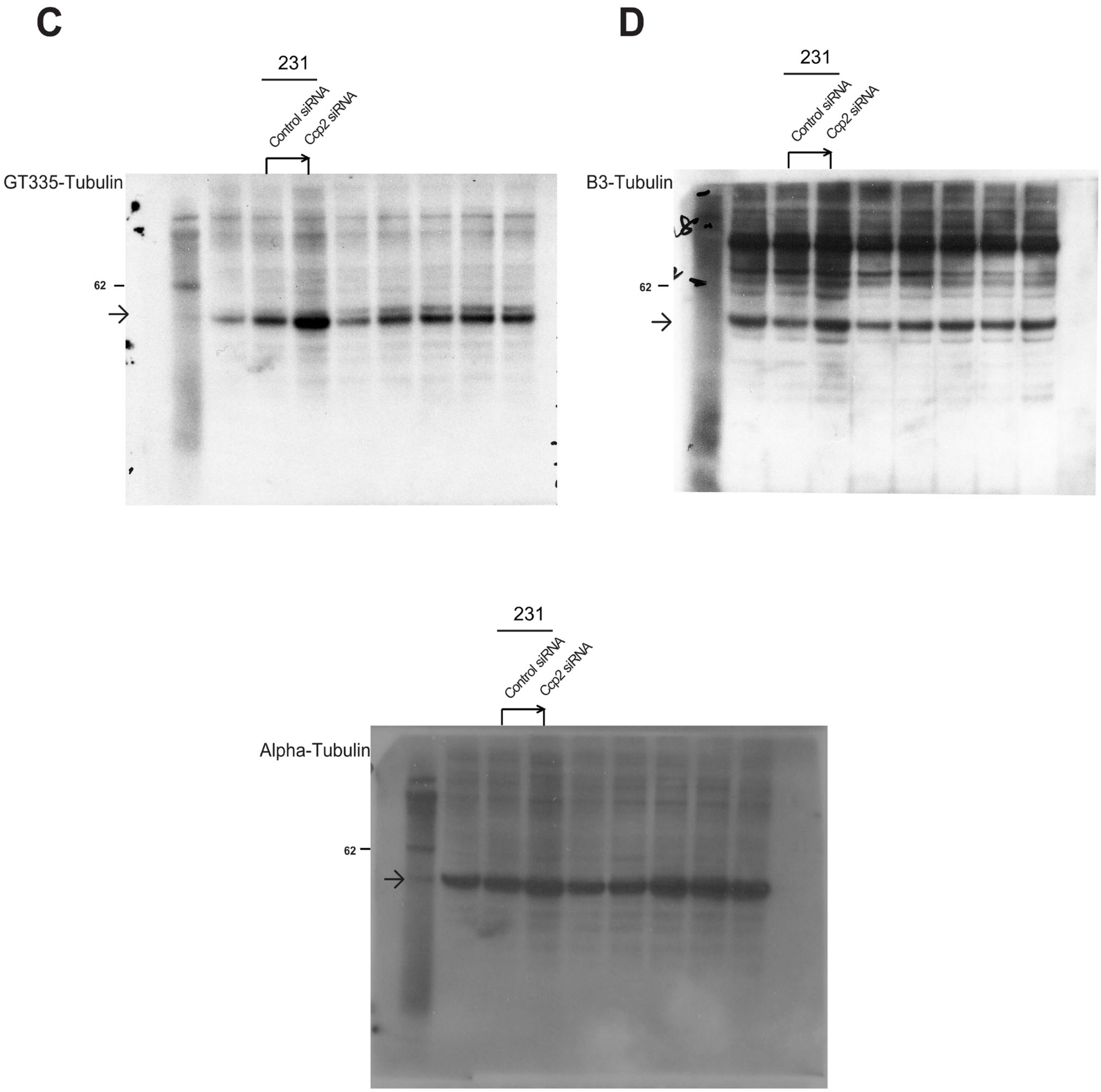

Supplementary Figure 14: Full-length immunoblots for Figure 1D: Immunoblot of GT335 (A, C) - and B3 (B, D) - polyglutamylated tubulin in CCP2 transient knockdown- MCF10A cells (A, B) and MDA-MB-231 cells (C, D), respectively. Control cells with scrambled siRNA and CCP2 siRNA transfected MCF-10A cells / MDA-MB-231 cells were assessed, and the knockdown efficiency was shown in Supplementary Figure 1. **A.**, **B.** Full-length western blots of CCP2 transient knockdown in MCF-10A cells are pictured. The left blots are probed for GT-335 and B3 polyglutamylated tubulin while the blots on the right are probed for beta actin. **C.**, **D.** Full-length western blots of CCP2 transient knockdown from MDA-MB-231 are pictured. The upper blots are probed for GT-335 and B3 polyglutamylated tubulin while the blots on the bottom is probed for alpha- tubulin.

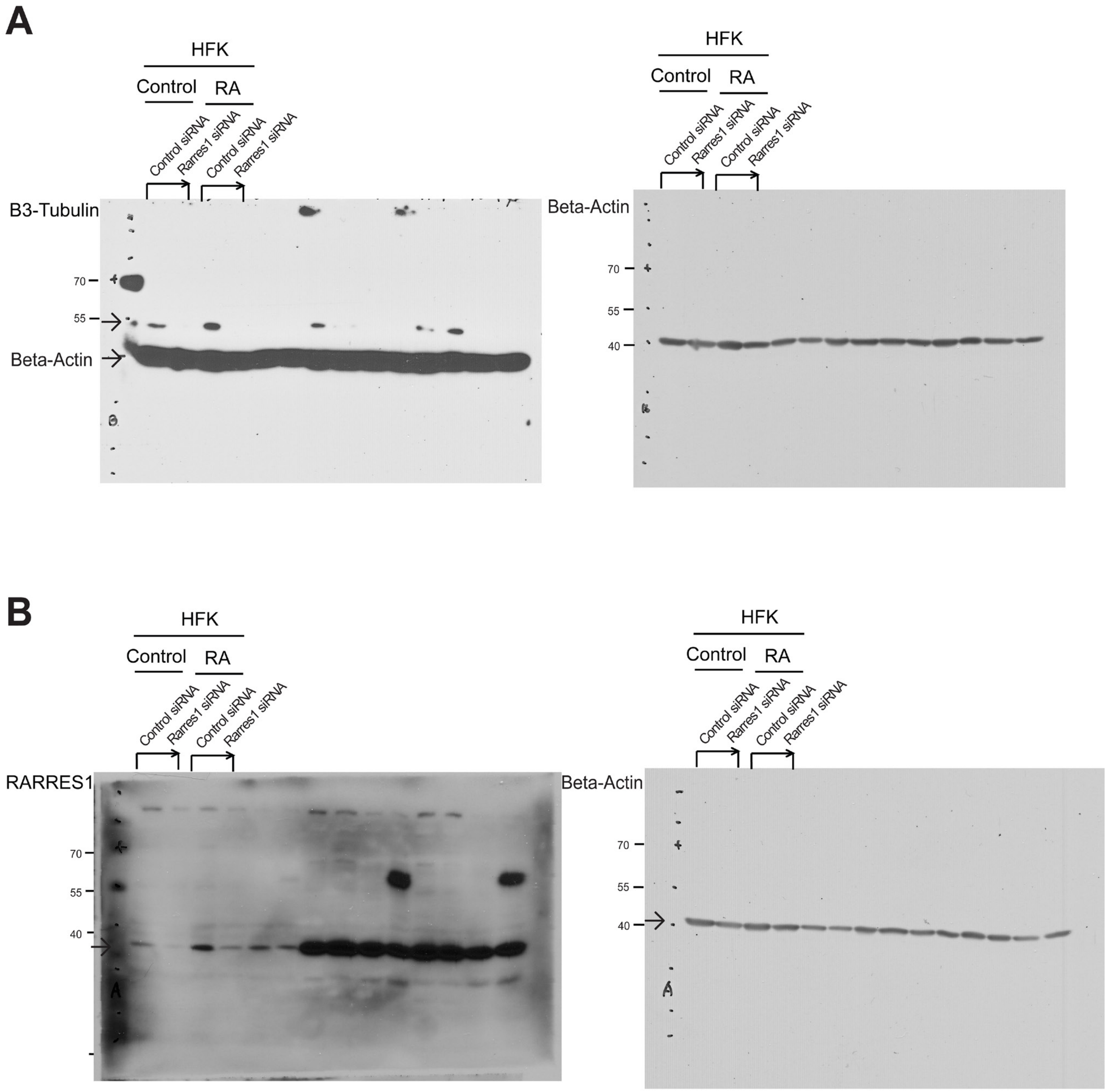
A
HFK
Control
RA
Control siRNA
Rarres1 siRNA
Control siRNA
Rarres1 siRNA
B3-Tubulin
70
55
Beta-Actin
HFK
Control
RA
Control siRNA
Rarres1 siRNA
Control siRNA
Rarres1 siRNA
Beta-Actin
70
55
40
B
HFK
Control
RA
Control siRNA
Rarres1 siRNA
Control siRNA
Rarres1 siRNA
RARRES1
70
55
40
HFK
Control
RA
Control siRNA
Rarres1 siRNA
Control siRNA
Rarres1 siRNA
Beta-Actin
70
55
40

C

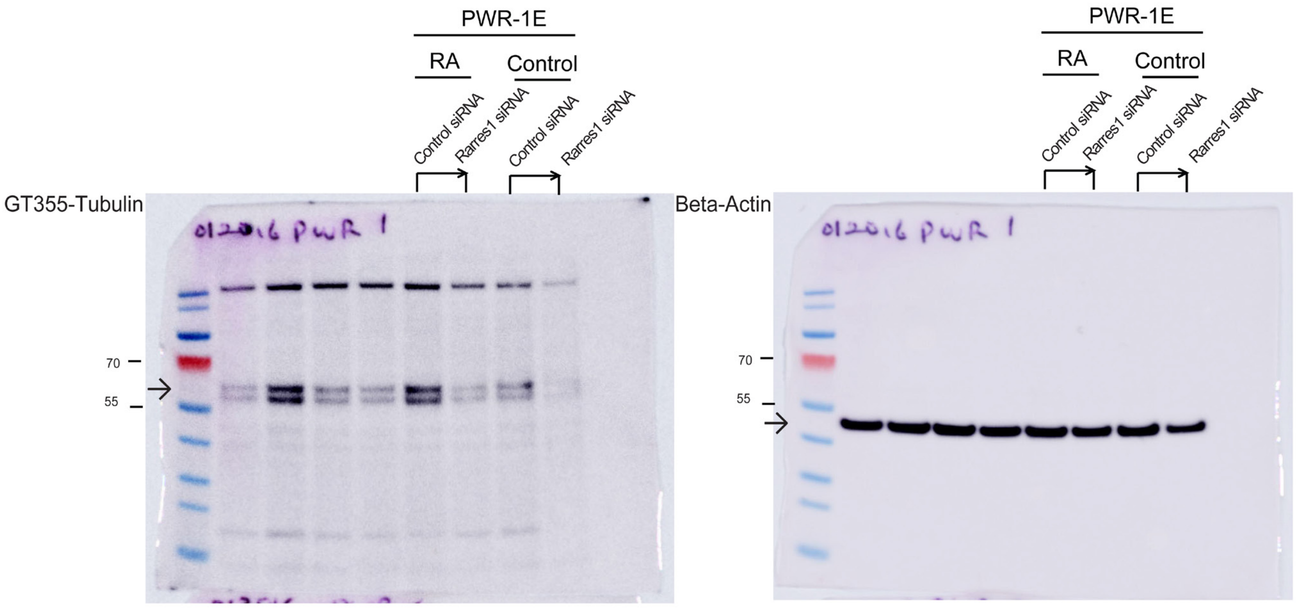

D

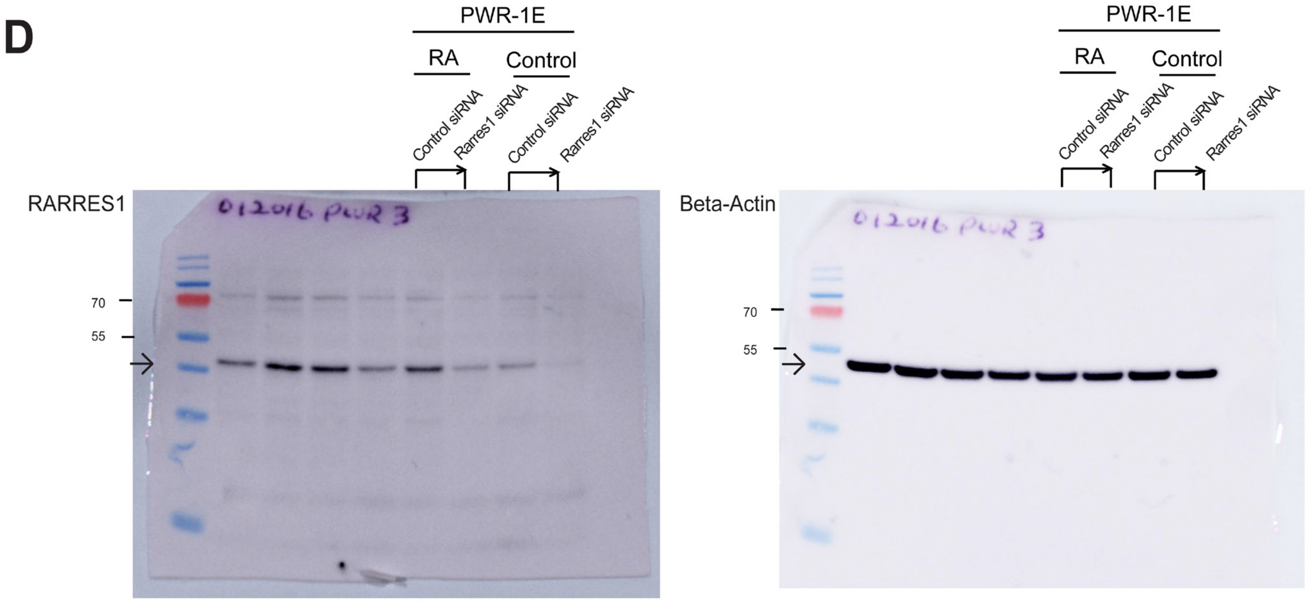

Supplementary Figure 15: Full-length immunoblots for Figure 1B and Supplementary Figure 3C: Immunoblot of polyglutamylated tubulin and RARRES1 after RARRES1 manipulation with or without retinoic acid in HFK (A, B) and PWR-1E cells (C, D). A., B. Full length immunoblots of B3 polyglutamylated tubulin and RARRES1 after RARRES1 manipulation with or without retinoic acid in HFK cells. The left blots are probed for B3 polyglutamylated tubulin and RARRES1 while the blots on the right are probed for beta actin. **C.**, **D.** Full length immunoblots of B3 polyglutamylated tubulin and RARRES1 after RARRES1 manipulation with or without retinoic acid in PWR-1E cells. The left blots are probed for GT-335 polyglutamylated tubulin and RARRES1 while the blots on the right are probed for beta actin.

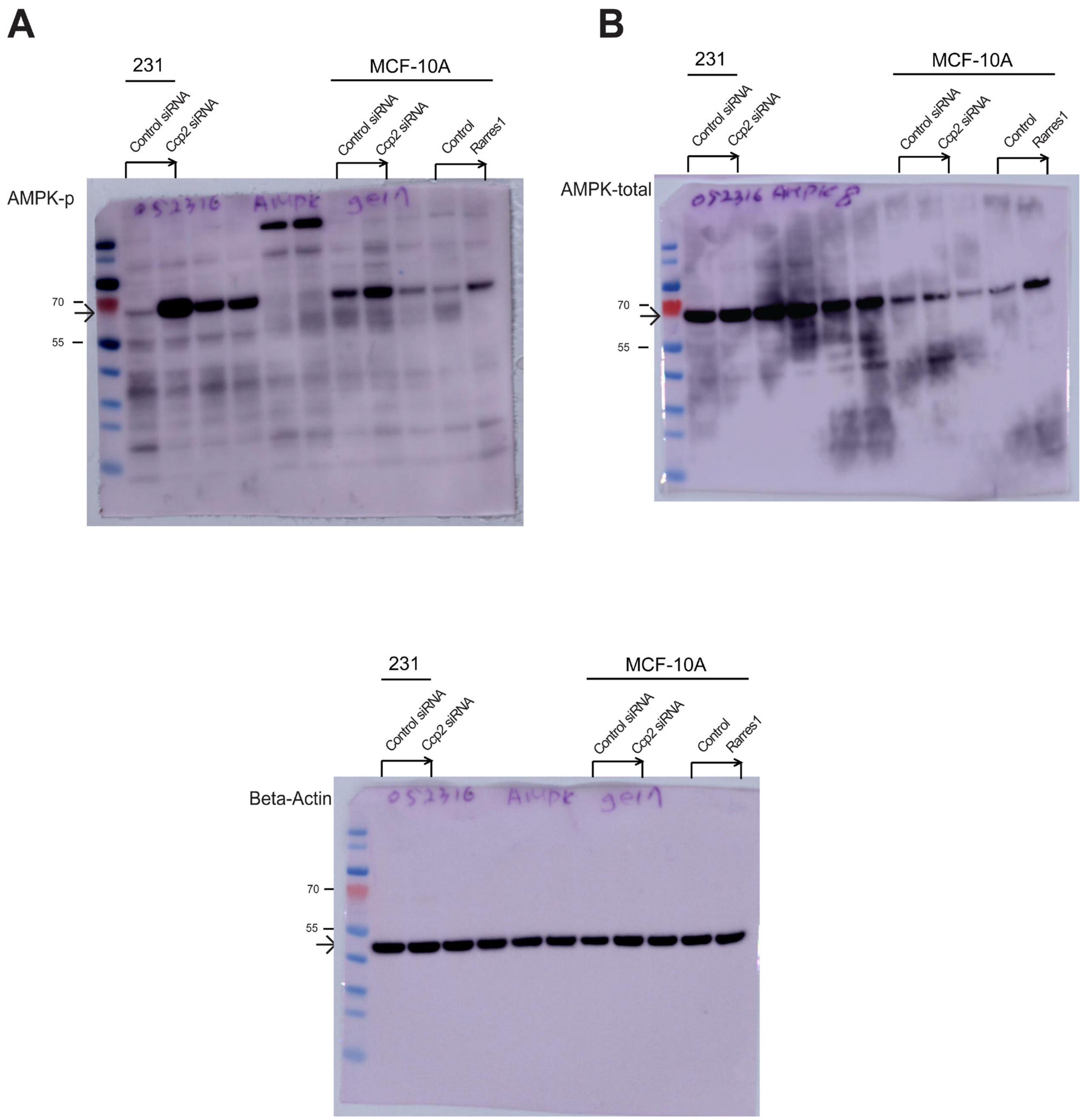

Supplementary Figure 16: Full-length immunoblots for Figure 6A: The blots displayed in this figure correspond to Figure 6A. Immunoblot for total and phosphorylated AMPK in MCF10A and MDA- MB-231 cells which RARRES1 or CCP2 was exogenously expressed or depleted. **A.**, **B.** Full- length western blots of RARRES1- overexpression and/or CCP2- transient knockdown in MDA- MB-231 and MCF 10A cells are represented. The left blot is probed for phosphorylated AMPK while the blot on the right is probed for phosphorylated AMPK. Alpha-tubulin was selected as a loading control in bottom side on this page.

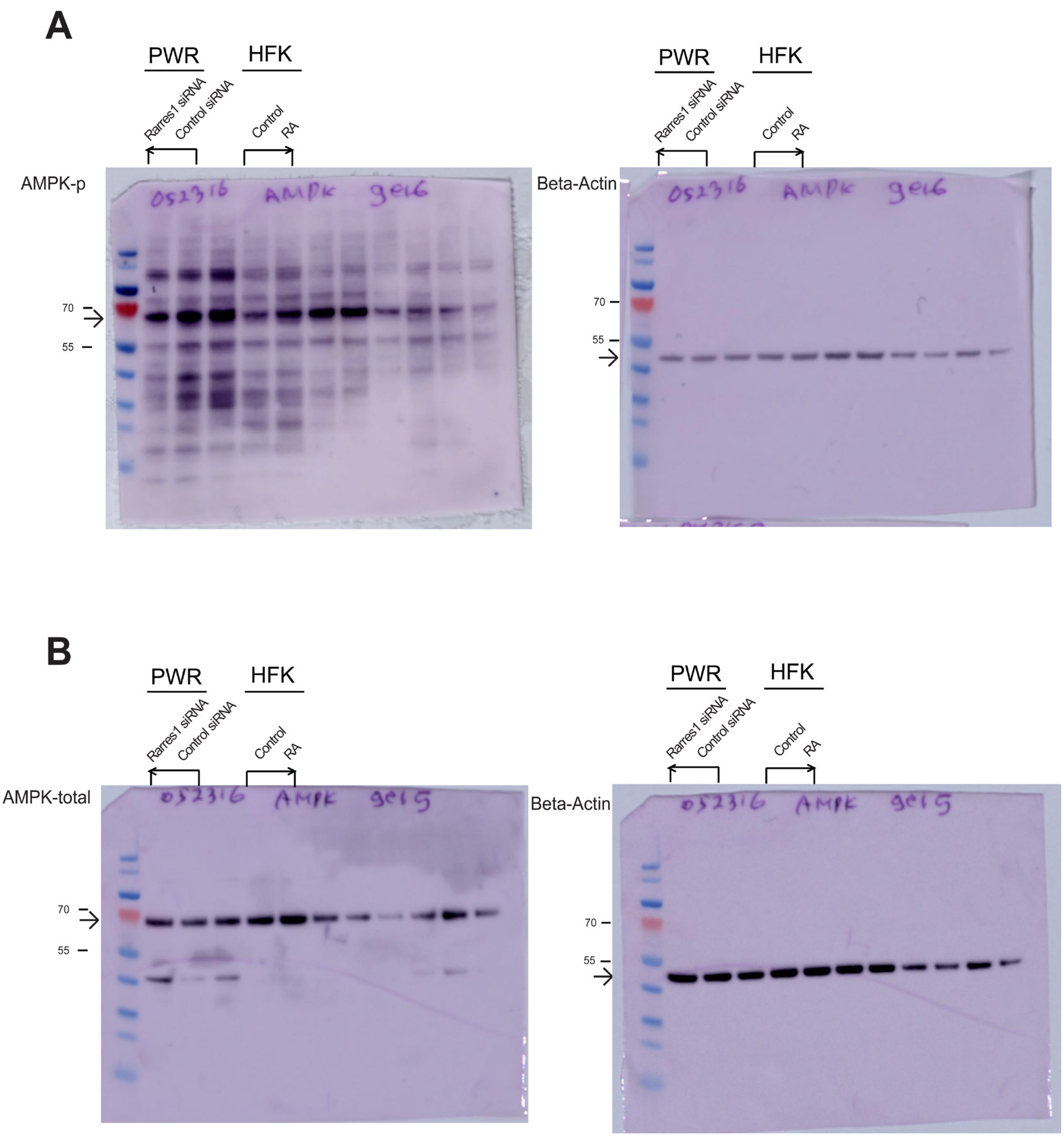
A
PWR
HFK
Rarres1 siRNA
Control siRNA
Control
RA
AMPK-p
70
55
Beta-Actin
70
55
B
PWR
HFK
Rarres1 siRNA
Control siRNA
Control
RA
AMPK-total
70
55
Beta-Actin
70
55

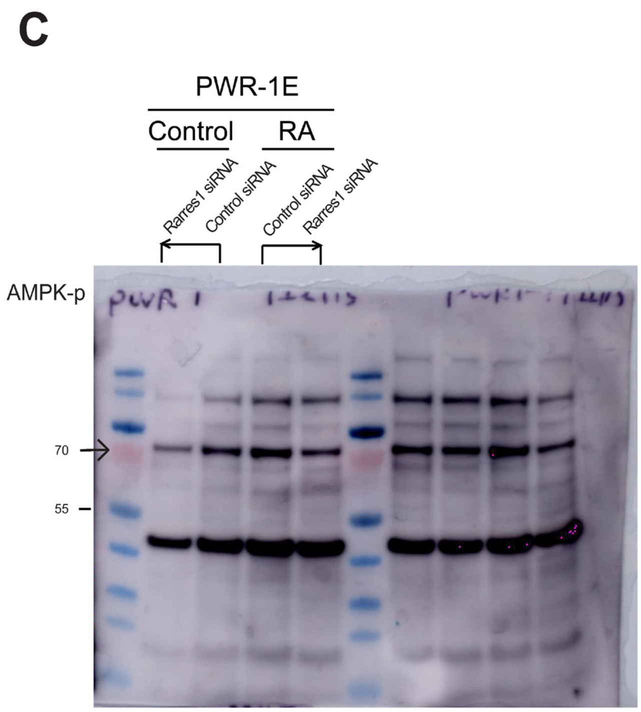

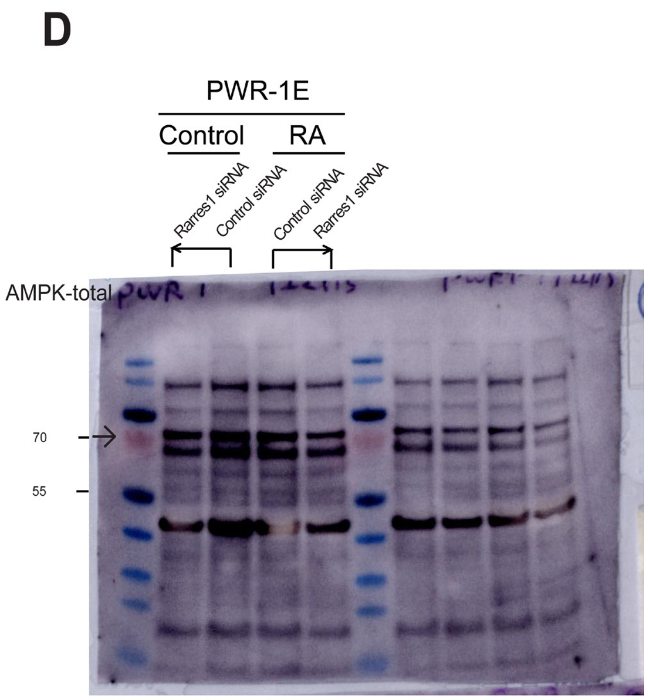

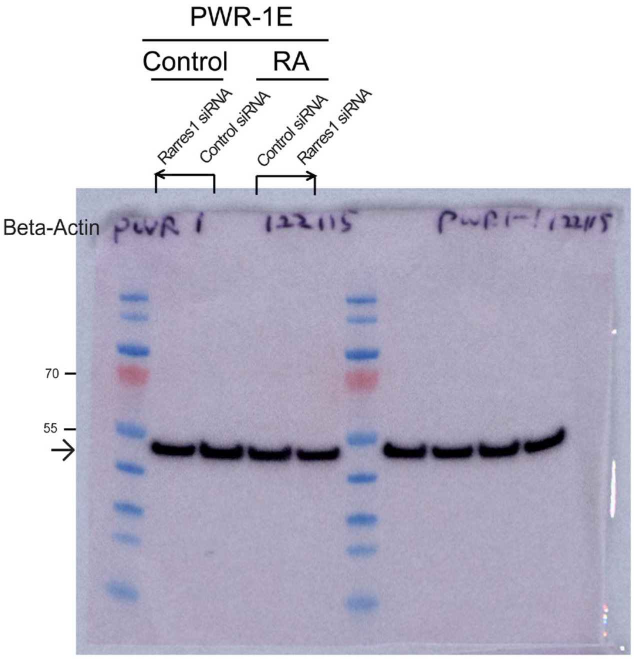

Supplementary Figure 17: Full-length immunoblots for Figure 6A and 6B: Immunoblot for total and phosphorylated AMPK in PWR-1E and HFK cells treated with RA or in which RARRES1 was depleted. A. Full-length western blots of RA treatment or RARRES1- transient knockdown in PWR-1E and HFK cells are represented. The left blot is probed for phosphorylated AMPK while the blot on the right is probed for beta actin. **B.** Full-length western blots of RA treatment or RARRES1- transient knockdown in PWR-1E and HFK cell-lines are represented. The left blot is probed for total AMPK while the blot on the right is probed for beta actin. **C.** Full-length Immunoblot for phosphorylated AMPK after RARRES1 depletion with or without retinoic acid in PWR-1E cells. **D.** Full-length Immunoblot for total AMPK after RARRES1 depletion with or without retinoic acid in PWR-1E cells.

A

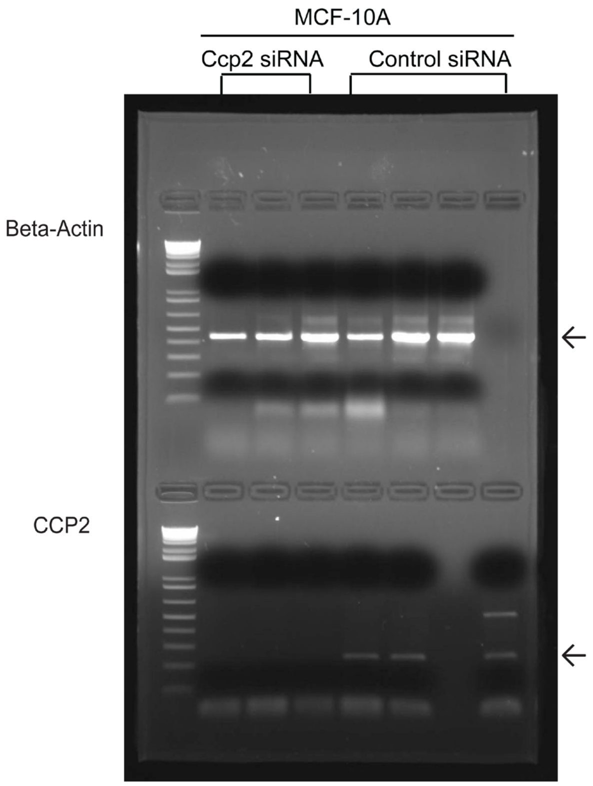
MCF-10A
Ccp2 siRNA
Control siRNA
Beta-Actin
CCP2

B

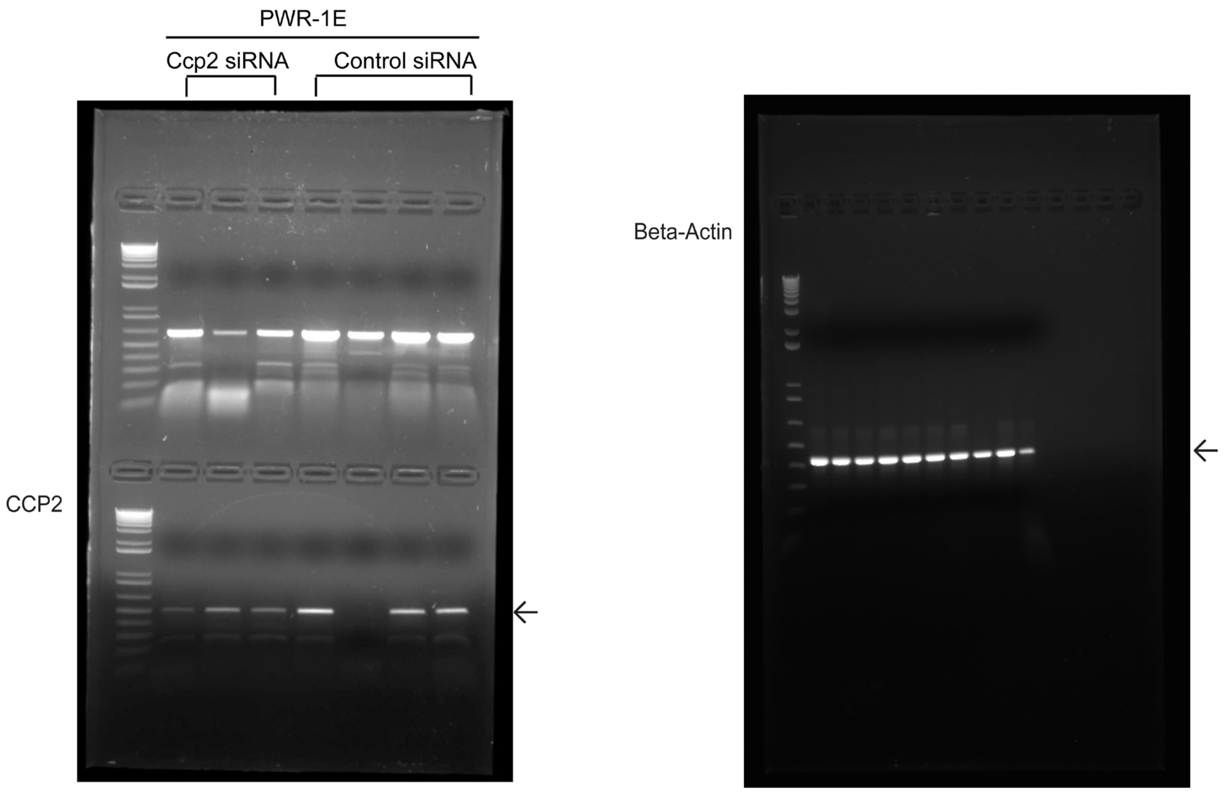
PWR-1E
Ccp2 siRNA
Control siRNA
CCP2
Beta-Actin

C

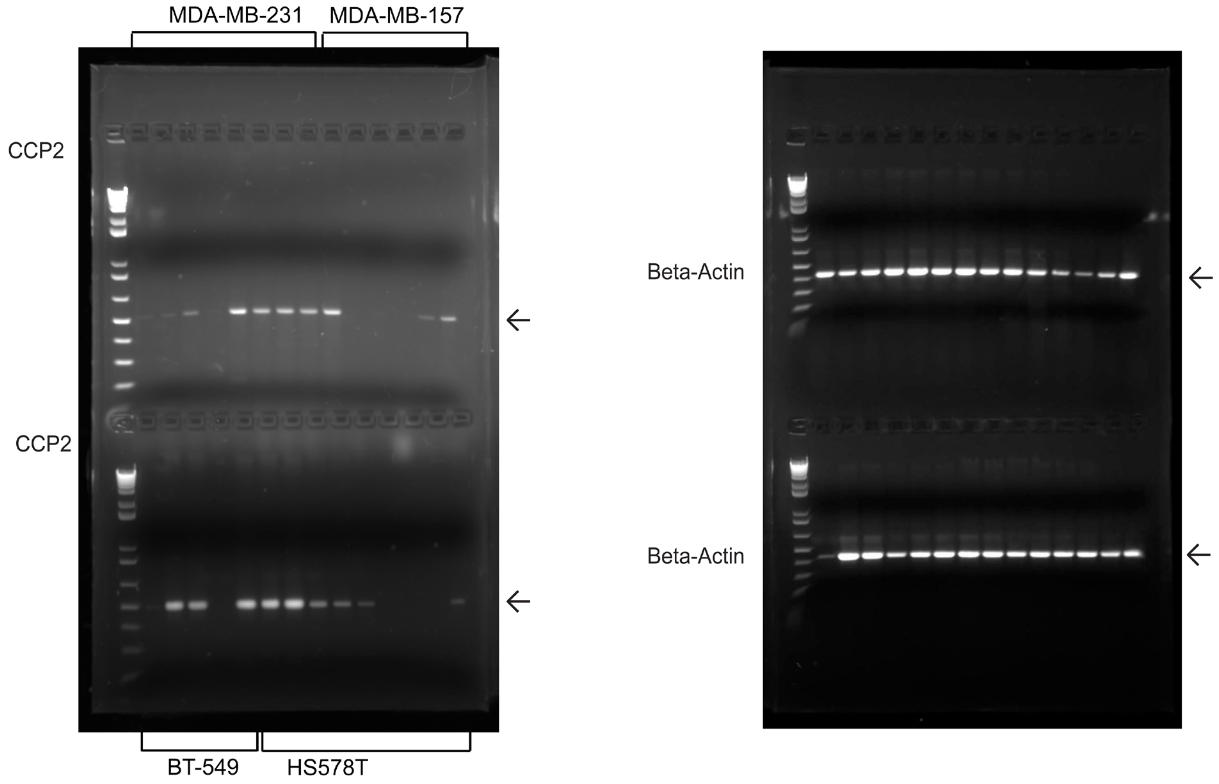

Supplementary Figure 18: Full-length PCR gels for Supplementary Figure 1C: CCP2 knockdown in non-tumorigenic cell lines (such as MCF10A and PWR-1E cells (A) and in tumorigenic cell lines, MDA-MB-157, MDA- MB-231, BT547, and HS578T cell lines (B) were validated through RT-PCR. These results were confirmed by qPCR (data not shown). Empty vector was also transfected with CCP2 siRNA to ensure the band is CCP2-specific. Beta-actin was used as an internal control. The lanes that are relevant to this study are highlighted in black borders.

Supplementary Table 1: Metabolites that were significantly changed in RARRES1 stable knockdown MCF-10A cells relative to the empty vector control cells

Name	Formula	FC	p.value	Query_ m/z	Exact_Mass
Gamma-glutamyl-Leucine ;Glutaminyl-Leucine	C11H21N3O4	1576.1	0.00587	258.1461	259.1532062
Hydroxyprolyl-Lysine	C11H21N3O4	1576.1	0.00587	258.1461	259.1532062
Isoleucyl-Gamma-glutamate ;Isole ucyl-Glutamine	C11H21N3O4	1576.1	0.00587	258.1461	259.1532062
PE(18:3(6Z,9Z,12Z)/P-18:0)	C41H76NO7P	1189.9	0.041291	724.529	725.5359403
Be ta-Citryl-L-glutamic acid	C11H15NO10	747.14	0.00015466	320.0613	321.0695957
PA(15:0/20:0)	C38H75O8P	736.15	0.00032223	689.5131	690.52
Tetrade canoyl-CoA	C35H62N7O17P3S	99.693	0.0008188	976.3062	977.3135738
Glutathione	C10H17N3O6S	53.788	0.0034724	306.0754	307.083806
PE(P-16:0e /0:0)	C21H44NO6P	40.302	0.0074738	436.2818	437.2906247
Cyclic ADP-ribose ;cADPR	C15H21N5O13P2	35.162	0.0028946	540.0538	541.0611088
MG(22:1(13Z)/0:0/0:0)	C25H48O4	24.889	0.00000981	411.3466	412.35526
12-Oxo-20-trihydroxy-le ukotriene B4	C20H30O7	18.6	0.001623	381.1902	382.1991533
PI(16:0/16:0)	C41H79O13P	17.591	0.013428	809.5158	810.5258291
UDP-glucose ; UDP-galactose	C15H24N2O17P2	16.756	0.00000178	565.0477	566.0550204
PG(14:1(9Z)/22:1(11Z))	C42H79O10P	9.7442	0.0005794	773.5334	774.54
Uridine diphosphate-N-ace tylglucosamine	C17H27N3O17P2	7.0465	0.00032716	606.0746	607.0815695
PE(18:3(9Z,12Z,15Z)/16:0)	C39H72NO8P	6.2775	0.0000259	712.4928	713.4995548
Ce ramide (d18:1/16:0);N- Palmitoylsphingosine	C34H67NO3	4.9333	0.00038534	536.5036	537.512095
LysoPE(18:0/0:0)	C23H48NO7P	3.8169	0.0042671	480.3079	481.3168394
LysoPC(15:0)	C23H48NO7P	3.8169	0.0042671	480.3079	481.3168394
LysoPE(0:0/18:0)	C23H48NO7P	3.8169	0.0042671	480.3079	481.3168394
Isobutyryl-L-carnitine	C11H21NO4	3.4468	0.00042169	232.1553	231.1470582
Oxidize d glutathione	C20H32N6O12S2	3.2163	0.0019858	611.1444	612.1519619
Arginyl-Arginine	C12H26N8O3	0.43247	0.038042	331.2215	330.2127867
Asparaginyl-Tyrosine	C13H17N3O5	0.42834	0.038603	296.1236	295.1168207
Tyrosyl-Asparagine	C13H17N3O5	0.42834	0.038603	296.1236	295.1168207
Calcitroic acid (D3)	C23H34O4	0.41101	0.044502	375.2512	374.2457096
N(6)-Me thyllysine	C7H16N2O2	0.40458	0.045992	161.1287	160.1211778
20:0-18:1-PS	C44H84NO10P	0.30762	0.0039426	816.5755	817.5832844
20:2-18:2-MGDG	C47H82O10	0.077855	0.0073501	805.5797	806.5907989
20:1-18:3-MGDG	C47H82O10	0.077855	0.0073501	805.5797	806.5907989
18:0-18:2-PE	C41H78NO8P	0.022421	0.0030813	742.5394	743.546505

The table lists features that were significantly altered in the KD cells via multivariate data analysis. These were putatively identified by accurate mass based search using Madison Metabolomics Consortium Database (MMCD) and Human Metabolome Database (HMDB). m/z denotes: mass in Daltons/charge; FC: Fold change; KD/C: (knockdown/control). P-values were calculated by student t-tests. The formula and exact mass of the metabolites were also included.
